# Distribution of microRNA profiles in pre-clinical and clinical forms of murine and human prion disease

**DOI:** 10.1038/s42003-021-01868-x

**Published:** 2021-03-25

**Authors:** Lesley Cheng, Camelia Quek, Xia Li, Shayne A. Bellingham, Laura J. Ellett, Mitch Shambrook, Saima Zafar, Inga Zerr, Victoria A. Lawson, Andrew F. Hill

**Affiliations:** 1 Department of Biochemistry and Genetics, La Trobe Institute for Molecular Science, La Trobe University, Bundoora, VIC Australia; 2 Department of Biochemistry and Molecular Biology, Bio21 Molecular Science and Biotechnology Institute, The University of Melbourne, Melbourne, VIC Australia; 3 Department of Mathematics and Statistics, La Trobe University, Bundoora, VIC Australia; 4 Department of Pathology, The University of Melbourne, Melbourne, VIC Australia; 5 Florey Institute of Neuroscience and Mental Health, The University of Melbourne, Melbourne, VIC Australia; 6 Department of Microbiology and Immunology, The Peter Doherty Institute for Infection and Immunity, University of Melbourne, Melbourne, VIC Australia; 7 Department of Neurology, University Medical Center Göttingen and German Center for Neurodegenerative Diseases, Göttingen, Germany; 8 Biomedical Engineering and Sciences Department, School of Mechanical and Manufacturing Engineering (SMME), National University of Sciences and Technology (NUST), Islamabad, Pakistan

**Keywords:** Neurodegeneration, Gene expression, Mechanisms of disease, Alzheimer's disease

## Abstract

Prion diseases are distinguished by long pre-clinical incubation periods during which prions actively propagate in the brain and cause neurodegeneration. In the pre-clinical stage, we hypothesize that upon prion infection, transcriptional changes occur that can lead to early neurodegeneration. A longitudinal analysis of miRNAs in pre-clinical and clinical forms of murine prion disease demonstrated dynamic expression changes during disease progression in the affected thalamus region and serum. Serum samples at each timepoint were collected whereby extracellular vesicles (EVs) were isolated and used to identify blood-based biomarkers reflective of pathology in the brain. Differentially expressed EV miRNAs were validated in human clinical samples from patients with human sporadic Creutzfeldt-Jakob disease (sCJD), with the molecular subtype at codon 129 either methionine-methionine (MM, *n* = 14) or valine-valine (VV, *n* = 12) compared to controls (*n* = 20). EV miRNA biomarkers associated with prion infection predicted sCJD with an AUC of 0.800 (85% sensitivity and 66.7% specificity) in a second independent validation cohort (*n* = 26) of sCJD and control patients with MM or VV subtype. This study discovered clinically relevant miRNAs that benefit diagnostic development to detect prion-related diseases and therapeutic development to inhibit prion infectivity.

## Abstract (web summary)

Cheng et al. present differentially expressed extracellular vesicle miRNAs in human clinical samples from patients who suffer from sporadic Creutzfeldt-Jakob disease. This study identifies biomarkers that can be used to detect prion-related diseases, providing insights into drug development for inhibiting prion infectivity.

## Introduction

Prion diseases (or transmissible spongiform encephalopathies) are an invariably fatal class of progressive neurodegenerative disorders, including sporadic Creutzfeldt-Jakob disease (sCJD) and Gerstmann-Straüssler-Scheinker (GSS) syndrome in humans^1^. The disease pathology is contributed by the conformational conversion of normal cellular PrP^C^ to the disease-associated isoform PrP^Sc^ which leads to the characteristic spongiform vacuolation in the brain and progressive loss of neurons. sCJD commonly presents as a dementing encephalopathy with myoclonus, cerebellar ataxia, and the typical duration of illness with a median survival of four months^2^. Clinical diagnosis of prion diseases is performed in an advanced stage of neurological decline, where affected individuals are often immobile and non-communicable. Confirmation of a diagnosis of prion diseases is either by undertaking a brain biopsy or autopsy. In order to improve diagnosis and intervention strategies for prion disease, we need to further understand the pathogenesis of prion diseases and the key regulators of the molecular processes during infection that drives the prion infectivity and disease spreading. Here, we seek to determine the molecular fingerprint of microRNA (miRNA) changes in the brain and serum of prion-infected mice that could be used as pre-clinical and clinical markers of sCJD.

miRNA molecules have been recognised as a key modulator in translational repression or mRNA degradation by binding to the 3′ untranslated region (UTR) of their target sites. In the context of prion diseases, animal models have revealed a list of miRNA signatures that is responsible for vital biological pathways including synaptic plasticity, neuronal development and cell survival^3–6^. Furthermore, miRNA found packaged in extracellular vesicles (EVs), such as exosomes, have been increasingly studied to determine whether they can serve as diagnostic biomarkers when isolated from blood^7–9^. Recently, isolation of neural-derived exosomes in blood^10^ suggests that exosomes derived from the brain may bypass the blood brain barrier (BBB). This may allow the ability to diagnose neurodegenerative diseases through a non-invasive blood test known as a liquid brain biopsy.

To investigate the temporal miRNA expression patterns in a specific brain region, we have utilised a well-characterised in vivo mouse model with the M1000 prion strain that is a mouse-adapted human strain isolated from a patient who died from GSS^11,12^. These infected mice develop first signs of neuropathology after the mid-incubation period, 13 weeks post-inoculation (wpi), in the thalamus and hippocampus which spreads to the occipital cortex and brain stem from 14 wpi with M1000-prions^3^. In this study, the thalamus brain region was collected from the M1000 infected mice and used to profile miRNA at timepoints representing the pre-clinical stage of disease (3 and 13 wpi, prion M1000 infected; week 3 (*n* = 6), week 13 (*n* = 5) and uninfected; week 3 (*n* = 6), week 13 (*n* = 6)) to identify possible pre-clinical miRNA markers. For comparison, tissues were also collected at clinical stage whereby the infected mice display symptoms (20 wpi, terminal, prion M1000 infected; terminal (*n* = 6) and uninfected; terminal (*n* = 5)). In addition, to identify potential pre-clinical blood-based biomarkers for sCJD, we also profiled EVs isolated from matching serum to identify the temporal relationship between miRNA expression changes in the thalamus and serum EVs across the course of the disease and how these changes are involved in the pathogenesis of prion diseases. The serum biomarkers could be used to develop a method to monitor the stage of disease progression in sCJD patients and assist in therapeutic treatment and trials.

Statistically significant miRNA identified in the M1000 infected mouse model at pre-clinical and clinical timepoints were selected for validation in a set of human clinical samples collected from sCJD patients and controls. Prion diseases include three forms: sporadic, familial and acquired by an infection which can display different pathological features. While the incubation period or pre-clinical period may be long (up to four decades) sCJD can progress very rapidly upon clinical diagnosis. Codon 129 of the prion protein gene (PRNP) is the site of a common methionine (M)/valine (V) polymorphism which is associated with certain phenotypes of human prion diseases such as sCJD. In the Caucasian population, 52% of individuals are M homozygous (MM), 36% are heterozygous (MV) and 12% are V homozygous (VV)^13^. The mean onset of the disease with those who are MM homozygous or MV heterozygous is 65 years and the average clinical duration is four months with a range of 1–18 months. Those who are V homozygous have been observed to have a clinical duration of 3–18 months where the mean age of onset to be between 41 and 81 years (reviewed in ref. ^14^).

A non-invasive blood test would be an invaluable tool for sCJD as it is typically a difficult and rare condition to diagnose. Patients are often misdiagnosed due to poor clinical measures and lack of effective systemic drug therapies. The miRNA panels identified in this study identified potential biomarkers for early diagnosis and to improve the outcomes of patients with suspected sCJD (rapid progressive dementia) that has a short clinical duration.

## Results

### Pathology associated with prion M1000 infected mice

**Fig. 1:**
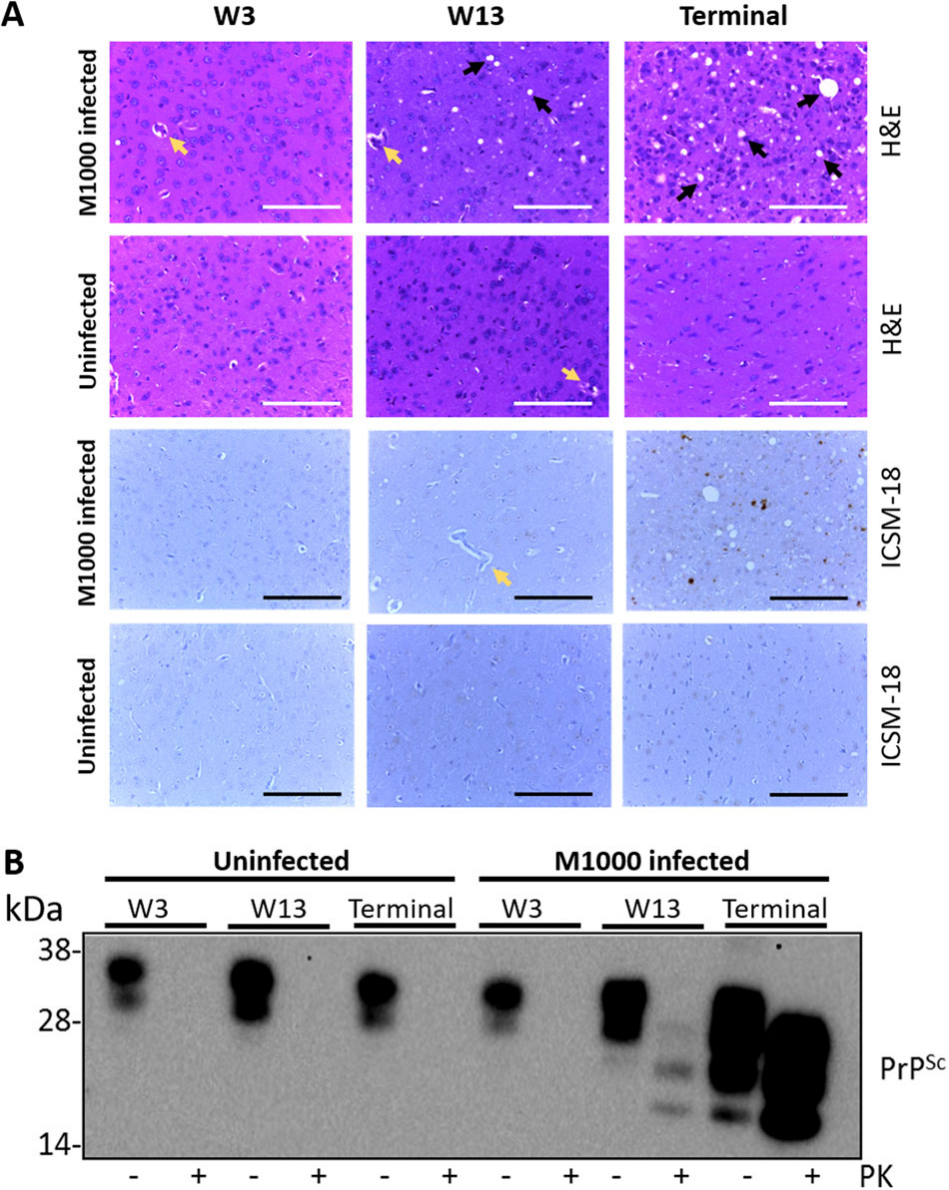
Pathology associated with prion M1000 infected mice. **A** Histology was performed using haematoxylin and eosin staining and ICSM-18 on thalamus brain sections of uninfected and prion-infected M1000 infected mice prepared at week 3 (W3), week 13 (W13), and when the mice manifested clinical signs of prion disease (terminal stage). Black arrows indicate prion-associated spongiform changes. Yellow arrows are present to differentiate from other features such as blood vessels. Images are taken at ×40 magnification. The scale bars shown are 100 μm. **B** The detection of PrP^Sc^ by Western blotting analysis of thalamus brain sections of uninfected and prion M1000 infected mice treated with proteinase K (PK, 100 μg/ml Proteinase K and 0.05% SDS for 1 h at 37 °C). Representative images of *n* = 4–5 per group.

A previously established in vivo model of prion disease with the M1000 prion strain was employed in this study^3,11^. Here, the progression of neuropathological changes was assessed over the time course starting from 3 wpi to the end-stage of disease (S1 Table). The histology images of the thalamus region from control (uninfected) and prion M1000 infected mice are shown in Fig. 1A. Consistent with the results from Brazier et al.^3^ spongiform change was first observed in the thalamus of prion M1000 infected mice at week 13 after inoculation. At the terminal stage, the spongiform change in the thalamus progressed to severe vacuolation and as reported previously this brain region showed prominent plaque deposition^3,11^. Analysis by western immunoblot showed PrP^Sc^ first detectable at 13 wpi with abundance increasing thereafter (Fig. 1B, Supplementary Fig. 1).

### miRNA expression pattern across different time points in the thalamus and serum

**Fig. 2:**
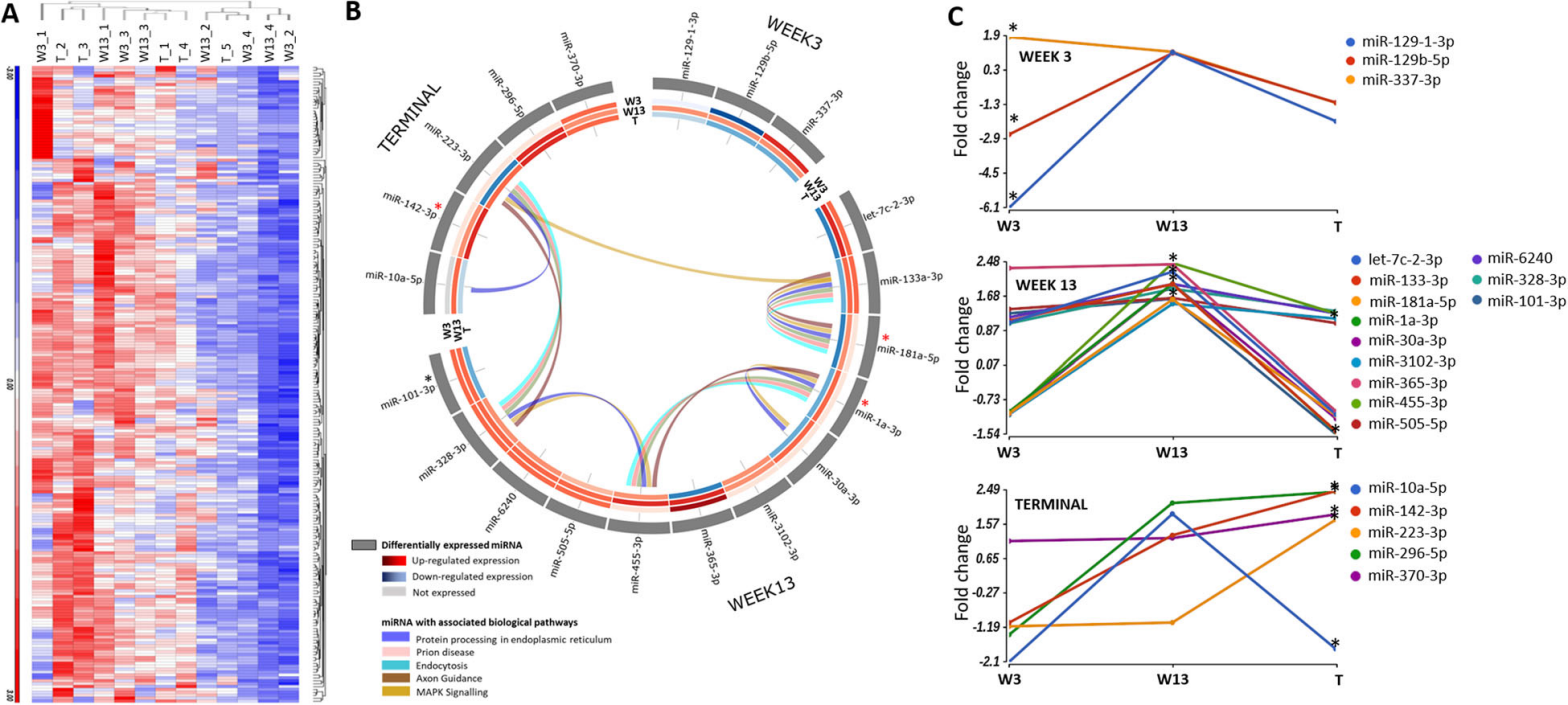
Expression of miRNA detected in the thalamus of prion M1000 infected and uninfected mice at week 3, 13 and terminal stage. **A** Hierarchical clustering of abundantly expressed miRNA (min > 10 RPM across in all samples) in M1000 infected mice at week 3, 13 and terminal stage. **B** Circos plot depicting the significant DE miRNAs (min > 10 RPM across all samples, ANOVA *p*-value < 0.05, corrected with Benjamini-Hochberg, fold change < −1.5 and >1.5) in M1000 infected mice compared to uninfected mice. The outermost track shows miRNAs that are DE in week 3, week 13 and terminal stage of disease. The red asterisks indicates those also found DE in serum EVs. The inner tracks illustrate expression heat map across all miRNAs in the time course study. The expression levels are represented in Log2 fold change. The ribbons inside the circos plot represent biological pathways the miRNA is involved in including prion diseases, MAPK (mitogen-activated protein kinase) signalling, protein processing in endoplasmic reticulum, endocytosis and axon guidance. **C** Longitudinal analysis of the DE miRNAs across week 3, 13 and terminal stage. *indicates the time-point which the miRNA was found to be significantly DE (ANOVA *p*-value < 0.05). Prion M1000 infected; week 3 (*n* = 4), week 13 (*n* = 4) and terminal (*n* = 5). Uninfected; week 3 (*n* = 5), week 13 (*n* = 5) and terminal (*n* = 5).

After confirming the neuropathology in the thalamus of prion M1000 infected mice from 13 wpi, small RNA sequencing was performed to assess miRNA expression patterns across three-time points which include 3, 13 and terminal wpi (Supplementary Table 1). The average amount of reads obtained per sample after quality trimming was 1.41 million reads with an average read length of 33 nucleotides (nt). Read length cut off was set at 18 nt with the Ion Torrent-based algorithm per-base quality score of greater than Q20 which is a Phred-like method to predict the probability of correct base calls at 99%. After alignment to the mouse genome (average of 95.16% alignment) and mapping to miRbase v21, 1907 miRNAs were identified in the entire dataset and the overall expression pattern was observed upon performing the principal component analysis (PCA, Supplementary Fig. 2). Abundantly expressed miRNA with a minimum of 10 Reads Per Million (RPM) across all samples, a total of 219 miRNAs, was visualised in a hierarchical clustering map to observe global miRNA changes in the thalamus across prion M1000 mice (Fig. 2A, Supplementary Table 2). Figure 2A revealed that the expression levels of these abundantly expressed miRNAs were different among samples across the time course. The expression of some miRNAs were not consistent and changed earlier or later, in particular at the terminal stage, amongst individual mice within the same time point. All mice were confirmed to be PrP^Sc^ positive by western blots at terminal however, variation may be due to longer incubation periods as terminal mice were culled between 18–22 weeks post infection (SD = 1.47 wpi; Supplementary Table 1). This may allow for further disease progression in individual mice. Nonetheless, statistically significant differentially expressed (DE) miRNA (min > 10 RPM across all thalamus samples, M1000 infected V’s uninfected at all timepoints, *p*-value < 0.05, corrected with Benjamini-Hochberg, fold change < −1.5 and >1.5) at each time point (Supplementary Table 3) were identified and represented in a circos plot (Fig. 2B). The result from Fig. 2B highlighted a total of 20 miRNAs (*p*-value < 0.05, corrected with Benjamini-Hochberg, fold change < −1.5 and >1.5) that were DE in this time course study; including three miRNAs (mmu-miR-129-1-3p, mmu-miR-129b-5p, mmu-miR-337-3p) that were identified at 3 wpi, 12 miRNAs (mmu-let-7c-2-3p, mmu-miR-133a-3p, mmu-miR-181a-5p, mmu-miR-1a-3p, mmu-miR-30a-3p, mmu-miR-3102-3p, mmu-miR-365-3p, mmu-miR-455-3p, mmu-miR-505-5p, mmu-miR-6240, mmu-miR-328-3p and mmu-miR-101-3p) at 13 wpi, and six miRNAs were identified in the terminal stage (mmu-miR-101-3p, mmu-miR-10a-5p, mmu-miR-142-3p, mmu-miR-223-3p, mmu-miR-296-5p and mmu-miR-370-3p) where mmu-miR-101-3p was found to be statistically significantly DE at both 13 wpi and terminal stage. Figure 2C demonstrates the longitudinal expression changes of these DE miRNAs across the time points collected. The majority of miRNAs show dynamic expression changes during the progression of infection and the most statistically significant changes are at 13 wpi at which point pathology changes are observed (Fig. 1A, B, Supplementary Fig. 1). While miRNAs statistically significantly DE at the terminal stage show an increase in expression over time (except for mmu-miR10a-5p).

**Fig. 3:**
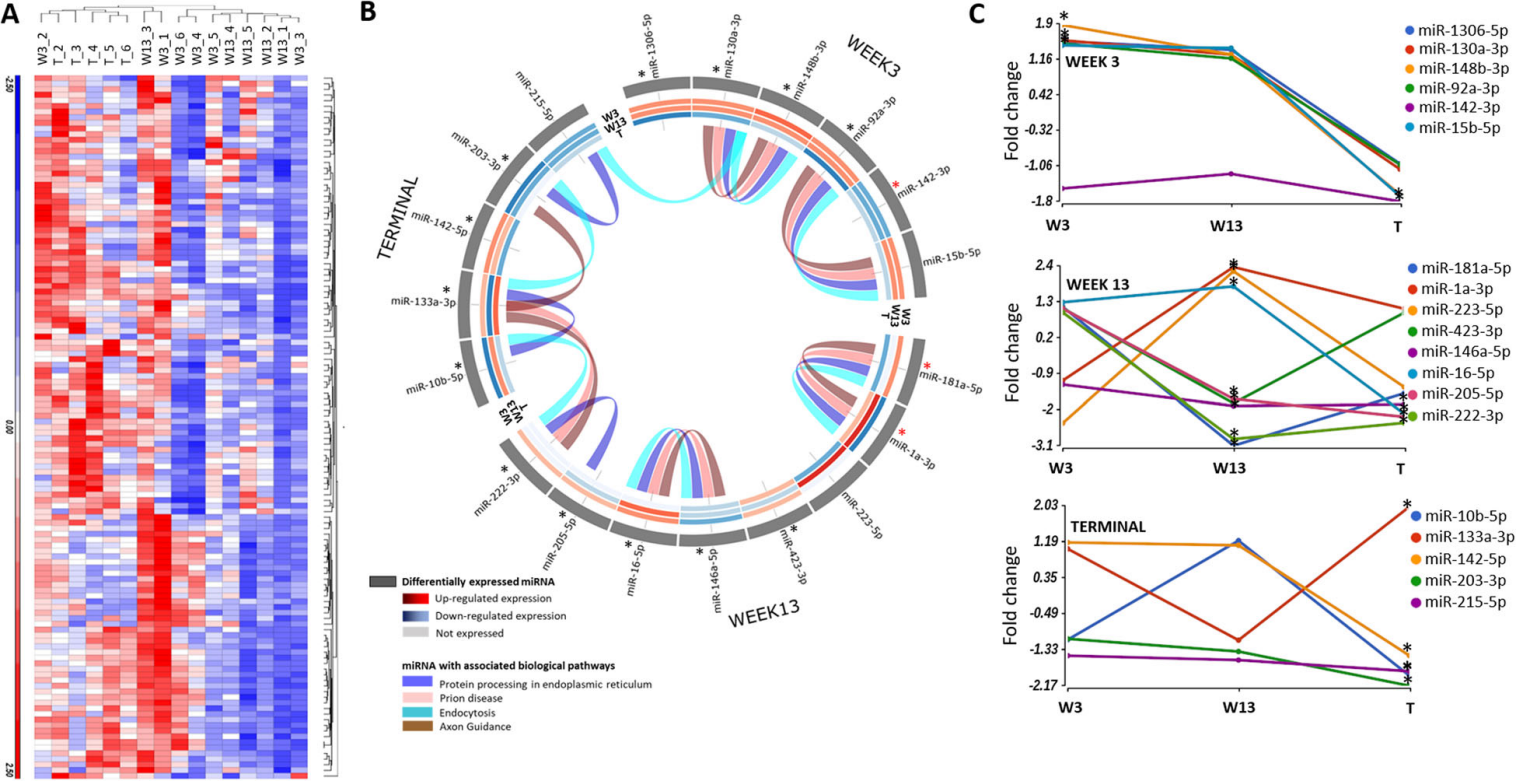
Expression of miRNA detected in serum EVs collected from prion M1000 infected and uninfected mice at week 3, 13 and terminal stage. **A** Hierarchical clustering of abundantly expressed miRNA (min > 10 RPM across in all samples) in prion M1000 infected mice at week 3, 13 and terminal stage compared to uninfected mice. **B** Circos plot depicting the significant DE miRNAs (min > 10 RPM across all samples, ANOVA *p*-value < 0.05, corrected with Benjamini-Hochberg, fold change <−1.5 and >1.5) in prion M1000 mice compared to uninfected mice. The outermost track shows miRNAs that are DE in week 3, week 13 and terminal stages of the disease. The red asterisks indicate the miRNAs also DE in the thalamus. All miRNAs with an asterisks (red or black) are those used in the human validation study. The inner tracks illustrate an expression heat map across all miRNAs in the time course study. The expression levels are represented in Log2 fold change. The ribbons inside the circos plot represent biological pathways the miRNA is involved in including prion diseases, protein processing in endoplasmic reticulum, endocytosis and axon guidance. **C** Longitudinal analysis of the DE miRNAs across week 3, 13 and terminal stage. *indicates the time-point which the miRNA was found to be significantly DE (ANOVA *p*-value < 0.05). Prion M1000 infected; week 3 (*n* = 6), week 13 (*n* = 5) and terminal (*n* = 5). Uninfected; week 3 (*n* = 6), week 13 (*n* = 6) and terminal (*n* = 6).

Serum EVs were also isolated from the same mice to determine whether a diagnostic read out of pre-clinical prion disease could be obtained from this longitudinal mouse study. Upon analysis of the small RNA sequencing data, global expression of abundantly expressed serum EV miRNA (125 miRNAs) of M1000 infected mice across the time course study was observed to be more reproducible across biological replicates (Fig. 3A, Supplementary Table 4) compared to what was observed in the thalamus tissues. miRNAs in the bloodstream are highly produced from all organs of the body and may have a long half-life that leads to a more stable expression across time that can be observed consistently across individuals^15^.

Figure 3B displays the DE miRNA (min > 10 RPM across all serum samples, M1000 infected vs uninfected at all timepoints, *p*-value < 0.05, corrected with Benjamini-Hochberg, fold change < −1.5 and >1.5, Supplementary Table 5) observed in serum EVs of M1000 infected mice compared to uninfected mice at each time point; including six miRNAs (mmu-miR-1306-5p, mmu-miR-130a-3p, mmu-miR-148b-3p, mmu-miR-92a-3p, mmu-miR-142-3p and mmu-miR-15b-5p) that were identified at 3 wpi, eight miRNAs (mmu-miR-181a-5p, mmu-miR-1a-3p, mmu-miR-223-5p, mmu-miR-423-3p, mmu-miR-146a-5p, mmu-miR-16-5p, mmu-miR-205-5p and mmu-miR-222-3p) at 13 wpi and 11 miRNAs (mmu-miR-142-3p, mmu-miR-15b-5p, mmu-miR-146a-5p, mmu-miR-16-5p, mmu-miR-205-5p, mmu-miR-222-3p, mmu-miR-10b-5p, mmu-miR-133a-3p, mmu-miR-142-5p, mmu-miR-203-3p and mmu-miR-215-5p) at terminal stage. Several miRNAs deregulated at clinical stage were also statistically significantly deregulated at pre-clinical stages which include mmu-miR-142-3p mmu-miR-15b-5p at 3 wpi and terminal stage, and mmu-miR-146a-5p, mmu-miR-16-5p, mmu-miR-205-5p and mmu-miR-222-3p at 13 wpi and terminal stage. Figure 3C demonstrates the longitudinal expression changes of serum DE miRNAs are also dynamic while many miRNAs show a decrease in expression over time. A number of these miRNAs found DE at two-time points of the study were selected for validation in human clinical samples to test their diagnostic potential with predicting sCJD (Supplementary Table 6). miRNAs selected were also crossed referenced with previously published small RNA sequencing data sequenced from human serum EV samples to ensure their abundancy in human samples^8,16^.

### Identification of prion diseases and their associated biological pathways

To gain insight into the biological pathways of DE miRNAs associated with the pre-clinical and clinical stage, enrichment analysis of multiple miRNA targets to all known (KEGG) pathways^17^ were performed (Fig. 2B). In the thalamus, at week 13, six deregulated miRNAs were found to have target in signalling pathways involved in MAPK Signalling, axon guidance, long-term potentiation, prion disease, protein processing in the endoplasmic reticulum and endocytosis (Fig. 2B). In serum EVs, the majority of deregulated miRNA at all timepoints were found to target genes involved in protein processing in the endoplasmic reticulum, prion disease, endocytosis and axon guidance as defined by the KEGG^17^ (Fig. 3B).

### Association of deregulated miRNAs in brain and serum EV samples

**Fig. 4:**
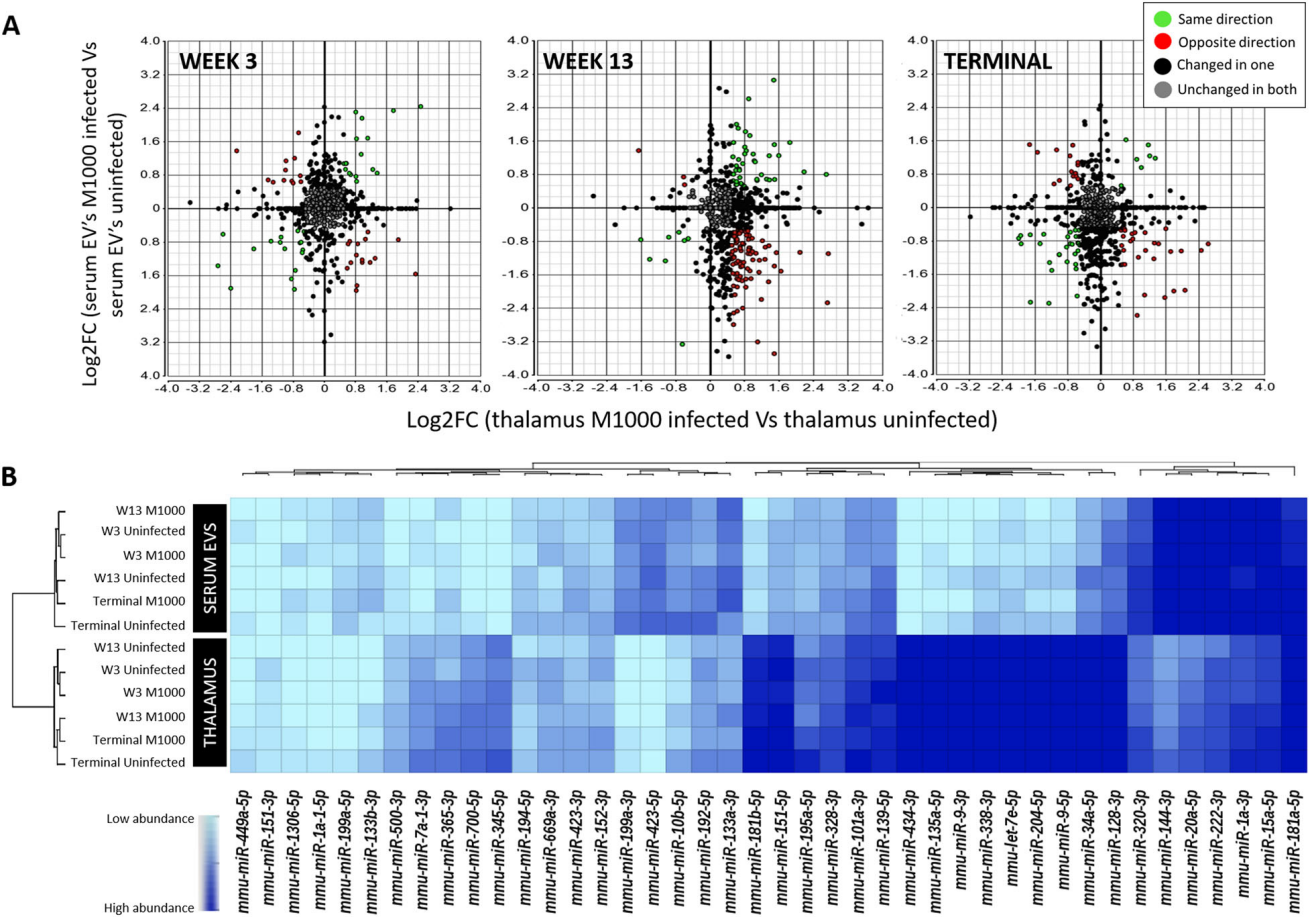
Direction of global miRNA expression changes in the thalamus and serum EVs of prion M1000 infected mice at week 3, 13 and terminal stage compared to controls. **A** ANOVA analysis was performed on the normalised reads from both datasets (thalamus and serum EVs) and were plotted as Log2 fold change. miRNA found to display inverse expression in serum EVs and thalamus are indicated in red. Those found to display the same direction in expression (either upregulated or downregulated) in both sample types are indicated in green. miRNA found unchanged in both samples types are indicated in grey and those changed in one sample type are indicated in black. Thalamus prion M1000 infected week 3 (*n* = 4), week 13 (*n* = 4) and terminal (*n* = 5). Thalamus uninfected week 3 (*n* = 5), week 13 (*n* = 5) and terminal (*n* = 5). Serum prion M1000 infected week 3 (*n* = 6), week 13 (*n* = 5) and terminal (*n* = 5). Serum uninfected week 3 (*n* = 6), week 13 (*n* = 6) and terminal (*n* = 6). **B** miRNAs indicated in red and green from panels in (**A**) are displayed in a heat map to visualise the abundancy of their expression in the thamalus and serum EVs. Only miRNAs with a minimum of 5 RPM across all samples are shown here. Log_2_ Mean RPM of each miRNA was used for the heat map.

Three miRNAs (mmu-miR-1a-3p, mmu-miR-181a-5p and mmu-miR-142-3p) were found to be statistically significantly deregulated in M1000 infected mice compared to uninfected mice in both the thalamus and serum EVs rendering them ideal biomarkers to validate as a liquid brain biopsy as they may indicate the presence of neuropathology (Figs. 2B and 3B, red asterisks). The expression of mmu-miR-1a-3p was observed to be upregulated approximately twofold in both the thalamus and serum EVs at 13 wpi. While, mmu-miR-181a-5p was found to be upregulated in the thalamus but downregulated in serum EVs. The expression of mmu-miR-142-3p was found to be inversely proportional in the thalamus compared to serum EVs at the terminal stage of the disease. To visualise the global direction of expression between miRNA found in the thalamus and serum EVs, the fold changes (Log2) between M1000 infected compared to uninfected mice at all timepoints were plotted in Fig. 4A. While many miRNAs were found to display unchanged expression in both the thalamus region and serum EVs of M1000 infected samples compared to uninfected samples (Fig. 4, grey datapoints), other miRNAs were found to be changed in only one sample type and not the other (Fig. 4, black datapoints). Interestingly, there were other miRNAs other than mmu-miR-181a-5p and mmu-miR-142-3p that displayed an inverse expression between the two sample types (Fig. 4, red data points). This may allude to the role of an active mechanism that regulates shuttling and uptake of exosomal miRNA at the BBB leading to differential miRNA expression on either side of the BBB. At 13 wpi, there was an overall increase of upregulated miRNAs, compared to 3 wpi and terminal stage (Fig. 4, panel 1 and 3, lower right quadrant, red data points), in M1000 infected thalamus while the same miRNAs remained downregulated in the serum (Fig. 4, panel 2, lower right quadrant, red data points). This supports that pathological changes occurring at the thalamus at 13 wpi (Fig. 1) are also reflected at the transcriptional level (Fig. 4). The comparison of these samples also revealed several miRNAs that displayed the same direction of expression, either upregulated or downregulated, in both sample types (Fig. 4, green data points). Those found to be upregulated in both the brain and serum EVs of M1000 infected mice such as mmu-miR-1a-3p were ideal for the validation study (Supplementary Table 6) as they would be easily detectable by diagnostic qRT-PCR methods.

Highly abundant miRNAs (minimum read count of 10 RPM across all samples) that show dynamic expression changes between the thalamus and serum EV (green and red data points in Fig. 4A) were visualised in a heat map (Fig. 4B). The heat map indicates clusters of miRNA species that are expressed to a lower degree (10–100 RPM) in both the thalamus and serum EVs (as indicated by the light blue shades, Fig. 4B). Other clusters of miRNA are extremely abundant (>1000 RPM) in both the thalamus and serum EVs (as indicated by the dark blue shades, Fig. 4B). While, certain miRNAs show greater expression in the thalamus compared to serum EVs or vice versa. This heat map demonstrates the miRNA species that can be found in the brain and periphery at relatively high expression levels that are likely to elicit a biological effect^18^. Furthermore, miRNAs with high copy numbers would be ideal biomarkers for qRT-PCR molecular pathology assays.

### Human clinical validation of prion associated serum EV miRNA biomarkers for sCJD

Serum samples from human sCJD cases and controls (Table 1) were obtained from the National Reference Centre for Prion Disease, Germany^19^ which comprised of subjects who were MM homozygous (*n* = 14) and VV homozygous (*n* = 12) that were clinically diagnosed with sCJD (Table 1). The controls (*n* = 20) used were patients referred as ‘suspected sCJD’ in which diagnosis of CJD had been definitely excluded by neuropathology^20,21^. They were then given an alternative diagnosis after thorough follow-up examination which included genotyping. Controls were aged matched and also underwent genotyping. EV miRNA was extracted and analysed by qRT-PCR using the primers towards 17 DE miRNAs identified in pre-clinical and clinical M1000 infected mice (Figs. 2B, 3B, indicated by asterisks, and Supplementary Table 6). The miRNAs selected for validation consisted of those DE in the serum EVs of pre-clinical and clinical M1000 infected mice which also includes the 3 miRNAs found DE in both the thalamus and serum EVs.

**Table 1:** Demographics of human clinical patients by study group.

Codon 129-PrPres subtype	sCJD M/M	sCJD V/V	sCJD M/V	sCJD unknown	Controls (M/M or M/V or unknown)
*Training cohort*					
*n*	14	12	–	–	20
Female	5	7	–	–	8
Male	9	5	–	–	12
Mean age (years)	70·1	61.8	–	–	64.3
SD age (years)	8.4	10.0	–	–	14.7
Biochemical subtype (Type 1/Type 2)^a^	14/0	12/0	–	–	5/-
Clinical duration (months)	2–5	2–15	–	–	N/A
14-3-3 positive (WB or ELISA)^a^	9	6	–	–	N/A
*Validation cohort*					
*n*	15	3	1	1	6
Female	10	2	1	1	3
Male	5	1	0	0	3
Mean age (years)	68.4	67	56	70	62
SD age (years)	9.4	6	N/A	N/A	9.8
Biochemical subtype (Type 1/Type 2)^a^	Unknown	Unknown	Unknown	Unknown	Unknown
Clinical duration (months)	1–17	4.1–10	25	3	N/A
14-3-3 positive (WB or ELISA)^a^	13	2	–	–	N/A

^a^Information was not available where the numbers do not add to the total *n* number.

**Fig. 5:**
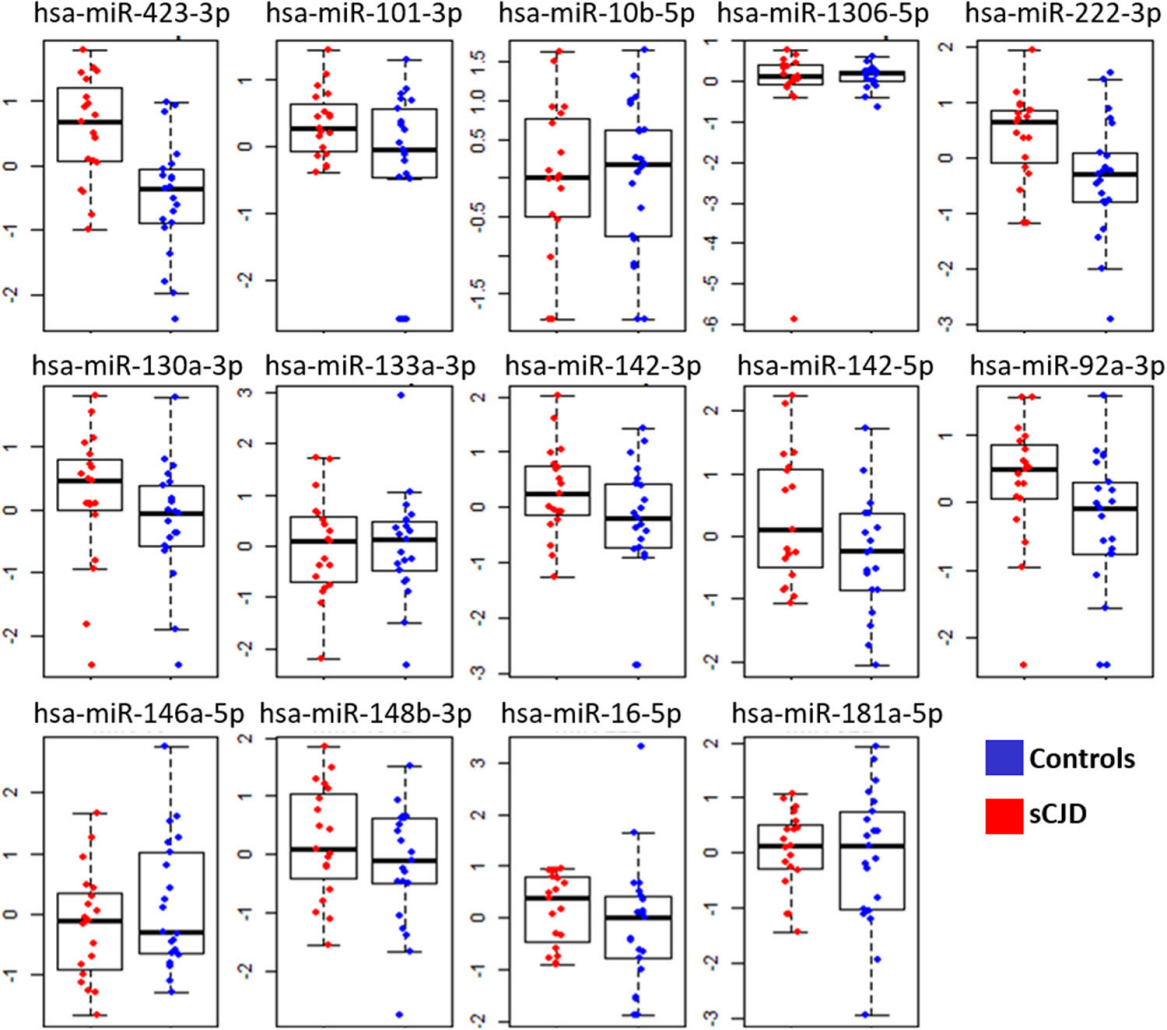
Box plots showing validated serum EV miRNA differentially expressed in controls and subjects with sCJD. Mean centred and scaled data were plotted between control and sCJD participants. control, *n* = 20 and sCJD, *n* = 26.

**Fig. 6:**
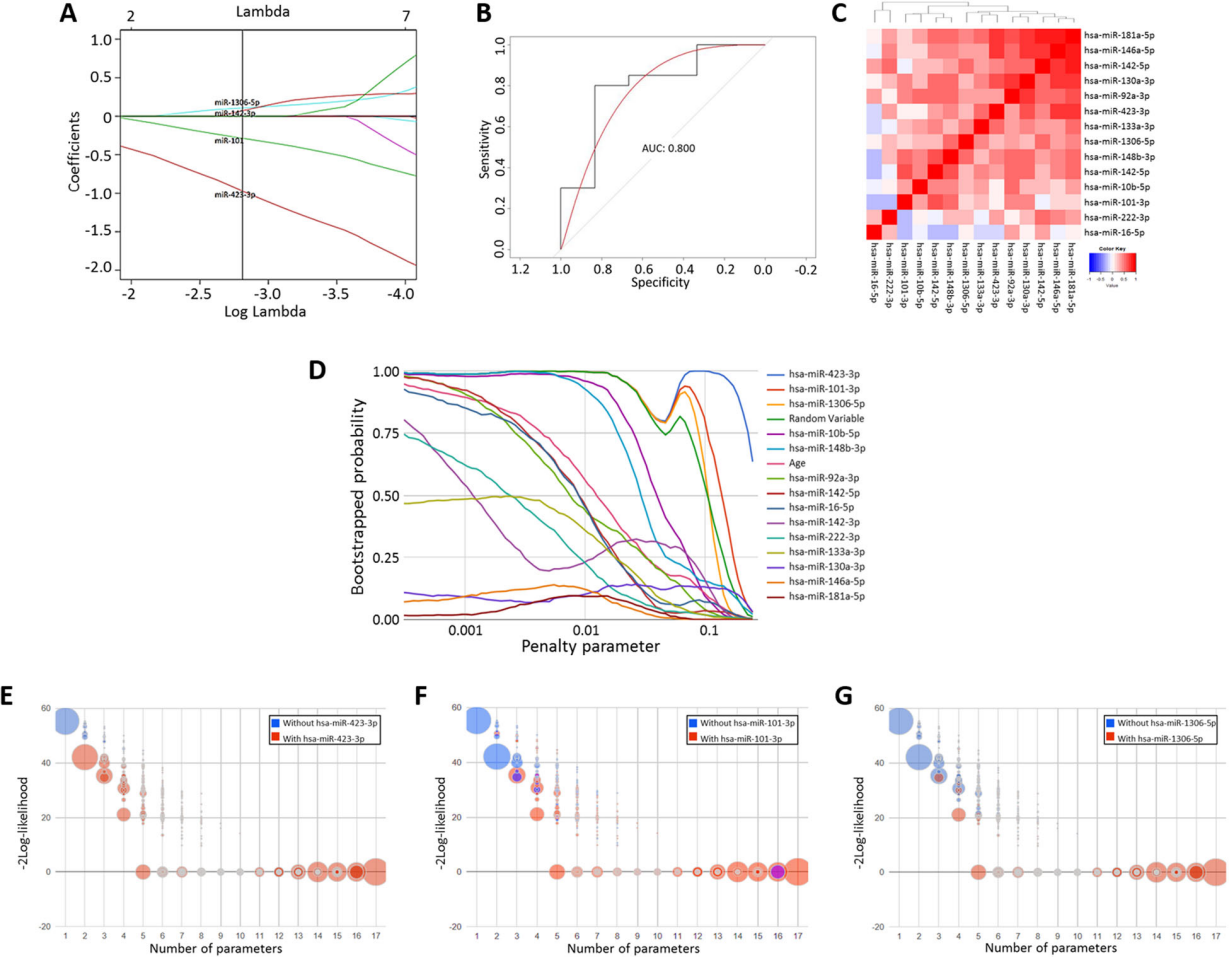
Performance of the LASSO model used to construct a miRNA signature to diagnose sCJD. **A** LASSO coefficient profiles of the 14 miRNAs including age and a random variable generated using the training cohort. Each curve corresponds to a miRNA. The vertical line is drawn at the value *λ* = 0.0794 chosen by 1000x cross-validation. **B** Receiver operating characteristic (ROC) curve for the performance of miRNAs and AUC values calculated using the validation set. **C** Correlation matrix heatmap of the 14 miRNAs from the training cohort, where each cell represents the Pearson correlation between the miRNA indicated in the corresponding row and columns. Dendrograms show the hierarchical clustering of the collinearity of the 14 candidate miRNAs. **D** The variable inclusion plots that indicate the three most important features above reference variable (RV). **E**–**G** Model stability bubble plots representing the stability of the model with the presence and absence of the strongest miRNAs in the model, hsa-miR-423-3p, hsa-miR-101-3p and hsa-miR-1306-5p. Each bubble represents a model and the colour of the bubble indicates whether (red) or not (blue) the model includes the particular miRNA. The size of the bubble corresponds to the probability of the model of the same size obtained from the ×1000 cross-validation.

Upon the acquisition of the qRT-PCR data, four miRNAs (hsa-miR-1a-3p, hsa-miR-203a-3p and hsa-miR-205-5p) were undetected across the majority of serum samples, likely due to lower abundancy in human serum or the volume collected compared to the mice discovery study, population diversity or inter-individual variation in the human population. Box plots of qRT-PCR data for each remaining miRNA markers (14 miRNA species) are stratified by the clinical classification are presented in Fig. 5. Overall, the majority of miRNAs analysed were downregulated in the sCJD group compared to the controls however, not statistically significant after multiple testing corrections. Finally, a LASSO model was applied on the whole dataset and the coefficient profiles of the 14 miRNAs and age at *λ* = 0.060 indicate four features (hsa-miR-423-3p, hsa-miR-101-3p, hsa-miR-1306-5p and hsa-miR-142-3p) that influence the response variable the strongest (Fig. 6A) providing a AUC of 0.924 (Fig. 6B). LASSO analysis correctly diagnosed 18 out of 21 sCJD subjects (85.7% sensitivity) and confirmed 17 out of 19 to be control subjects (89.5% specificity). This suggests that a panel of at least 4 miRNAs are required for classification. We performed analysis on the co-expression of all combinations of miRNAs but did not find improved accuracy above the final 4 combined miRNAs. A second cohort of samples was obtained and an independent validation study was performed on an additional 26 clinical samples (sCJD, *n* = 20 and controls, *n* = 6). The model was able to correctly confirm 17 out of 20 sCJD patients and four out of six controls. The resultant sensitivity was 85% and specificity at 66.7% with an AUC of 0.800 in the independent validation cohort. Specificity could be further improved upon the recruitment of further controls however, are currently difficult to access. The correlation matrix heatmap of the 14 miRNAs from the training cohort suggests that there are clusters of miRNAs that display a strong positive correlation and to a lesser extent, some negative correlations (Fig. 6C). Variable inclusion plot demonstrate which variables are important based on 1000 bootstrap sampling (Fig. 6D). Model stability plots (Fig. 6E–G) demonstrated the reliability of the final predictive model whereby the inclusion of up to 17 variables during bootstrap resampling displayed dominant models required the strongest 3 miRNAs (hsa-miR-423-3p, Fig. 6E, hsa-miR-101-3p, Fig. 6F and hsa-miR-1306-5p, Fig. 6G).

## Discussion

In order to investigate temporal-specific differences in expression accompanying disease progression, we examined all miRNA transcripts that were present in the thalamus over the course of prion infection using next-generation sequencing. Here, we reported the contribution of miRNAs to the establishment of prion disease by modulating gene expression post-transcriptionally, leading to the alteration of neuronal survival and signal transduction pathways.

At the earliest stage of infection, DE genes in the thalamus were found to be involved in prion diseases and axon guidance. This suggests that the earliest detectable miRNA profiles during early prion infection may trigger the initial aberrant deregulation of genes associated with neuronal damage which should be investigated using miRNA mimics and inhibitors in future studies. In addition, the miRNAs found to be involved in endocytosis and protein processing in the endoplasmic reticulum may deregulate target genes involved in endosomal sorting of proteins and RNA including exosomal content.

During the pre-clinical stage at week 13, a greater number of miRNAs were DE in the thalamus and serum while three miRNAs (mmu-miR-181a-5p, mmu-miR-423-3p and mmu-miR-101-3p) were found to be statistically significantly DE in both sample types of M1000 infected mice. This set of miRNAs are may be representative of pathological changes at week 13 in the brain and may serve as pre-clinical biomarkers. Spongiform changes were observed in the thalamus at week 13 and the expression changes suggested that cascade of intrinsic signalling events (MAPK signalling) was occurring in the infected neurons during these neuropathological changes. In the literature, various miRNAs have been shown to specifically target mRNAs that may be involved in prion disease pathology. mmu-miR-328, DE at week 13 and terminal, was previously shown to interact with the 3′ UTR of mRNA of β-secretase 1 (BACE1), which play an important role in the regulation of amyloid-β (Aβ) production through the cleavage of amyloid precursor protein^22,23^. Although some studies recently reported that PrP can interact with Aβ by acting as a neuronal surface receptor for oligomeric Aβ to mediate cytotoxicity, it remains unclear whether BACE1-specific miR-328 directly affects prion propagation through modulating BACE1^24–26^.

As the prion infection progressed to the terminal stage, five miRNAs were DE in the thalamus and serum EVs. However, several more miRNAs were found to be statistically significantly DE in serum EVs at both week 3 and terminal stage (mmu-miR-142-3p and mmu-miR-15b-5p) followed by week 13 and terminal stage (mmu-miR-146a-5p, mmu-miR-16-5p, mmu-miR-205-5p and mmu-miR-222-3p), potentially providing pre-clinical biomarkers for sCJD. Of these, differentially expressed miR-146a-5p was of relevance in this study. Majer et al.^27^ detected an incremental up-regulation of miR-146a-5p over the course of infection during the accumulation of prions in hippocampal neurons. At end-stage disease, miR-146a-5p was found to be upregulated in the frontal cortex and cerebellum of human post-mortem sCJD MM1 cases compared to sCJD VV2 cases and controls^28^. Another study performed at the terminal stage revealed a signature of miRNAs, including miR-146a-5p, expressed in the cortical region of mice infected with prion strains 139 A, ME7, and S15^29^. In this study, miR-146a-5p was found to be upregulated in the thalamus however, did not meet our stringent filter of minimum RPM for further analysis. Potentially, miR-146a-5p is not abundantly expressed in the thalamus of M1000 infected mice compared to other prion models used in the literature however, this may depend on the filters applied during bioinformatics analysis. In this study, we chose to focus on highly abundant miRNAs (defined as miRNAs with a minimum 10 RPM across all samples) to increase the confidence in the KEGG analysis and the likelihood of the contribution of DE miRNAs in biological processes. Nonetheless, miR-146a-5p was down-regulated at the pre-clinical stage of M1000 infected mice. As miR-146a-5p is a well-known immune regulatory miRNA and is commonly found in neuronal degeneration, miR-146a-5p may potentially serve as a general pre-clinical biomarker of neuroinflammation^30^.

In recent studies, the detection of let-7b, miR-130, miR-146, miR-223 and miR-23 from the current work has been highly associated with exosomes derived from neuronal cells and biological fluids^7,16,18,31,32^. These miRNAs have been implicated in the brain and plasma through the regulation of mRNA coding for various neuronal and developmental proteins. For example, the literature has revealed the clinical relevance of the interactions between miR-223-3p and its respective interacting mRNA coding for PRNP (prion protein) and the PRKACA (protein kinase A) gene^33^. The regulatory role of miR-223 promotes cellular differentiation and development in some cancers (stomach and ovarian cancer), leading to aggressive cancer growth and increased tumour burden^34–36^. The deregulation of miR-223 was observed at the later time points in this study, suggesting that miR-223 may play an active role in aggravating the disease progression during prion infection. It has been shown that the interaction of cellular prion protein with stress inducible protein not only activates protein kinase to transduce a survival signal, but also induces phosphorylation/activation of the MAPK signalling molecules to promote neuritogenesis^37,38^. The coordinated expression of the DE miRNAs in the thalamus may influence the disease progression through modulating genes involved in prion degeneration.

EVs may potentially migrate across the blood-brain barrier and exert biological effects on neurons by being internalised in the central nervous system^39,40^. The identification of these miRNAs from the brain and plasma suggested that EVs provide a means for trans-cellular communication between the brain and distant organs^7,41^. Figure 4A, B demonstrates that potentially only selective miRNA species have roles in both the brain and periphery, as not all miRNAs found in the brain were also detected in the serum. Brain-derived EVs isolated from post-mortem subjects diagnosed with Alzheimer’s disease were recently profiled for miRNA and correlated to serum EV miRNA profiles^42^. The study demonstrated while there was little correlation between serum EVs and brain-derived EV miRNA profiles, there were common miRNA species between the two tissue types. Furthermore, certain miRNAs species were found enriched in brain-derived EVs compared to miRNAs profiled from the whole brain. miRNAs enriched in brain-derived EVs may be selectively packaged and taken up by recipient cells to assist in the propagation of disease pathology. In a similar manner, abundantly expressed miRNAs are more likely to have a biological role compared to those present at low copy numbers^18^ or downregulated in a system. Further development of this present study could involve profiling the miRNA contained in brain-derived EVs of sCJD subjects or thalamus-derived EVs from the M1000 prion mice model. The ability to isolate exosomes from blood and correlating them with potential brain-derived signatures allows the possibility to detect highly relevant prion-associated miRNAs in the periphery for diagnostic testing.

The mouse model which propagates the M1000 prion strain was used in the discovery study to extrapolate potential miRNA biomarkers for testing in sCJD patients. While there may be inter-disease and region variability between M1000 prions and subtypes of human prion disease, the miRNAs identified are likely to alter common downstream biological pathways during the progression of this disease, irrespective of subtype. Recently, circulating miRNA serum profiles were identified from an elk prion model of chronic wasting disease (CWD). Infected elks were found to have deregulated hsa-miR-148a-3p, hsa-miR-186-5p and hsa-miR-30e-3p and hsa-miR-500a-3p, giving an AUC of 0.793 upon cross-validation for diagnosing CWD^43^. Slota et al. also validated their biomarkers in a second species, serum collected from 293 K scrapie infected hamsters, and combined commonly differentially expressed miRNAs in both species (elk and hamster) to identify a new miRNA signature (hsa-miR-103a-3p, hsa-miR-107, hsa-miR-99a-5p, hsa-miR-125b-5p and hsa-miR-100-5p) associated with both prion models. The diagnostic accuracy of their model upon validation with the same infected CWD and non-infected elk samples was 0.867. This further demonstrates that miRNA biomarkers may be useful to identify prion infection across several animal species. Upon comparison between our two studies, the miR-148 family of miRNAs were deregulated in both studies however, at different directions in serum depending on whether the animal exhibited pre-clinical or clinical signs of infection. Dissimilarities observed between our studies may be due to differential profiles obtained through isolating total circulating serum miRNA versus enriching for serum EV miRNA.

Despite the inter-disease and species variability, pre-clinical miRNA candidates from this mouse model were demonstrated to classify sCJD subjects from a human clinical cohort with an AUC of 0.800. In comparison, Norsworthy et al. performed miRNA profiling on whole blood samples collected from a cohort of sCJD patients which resulted identifying 3 miRNAs (hsa-let-7i-5p, hsa-miR-16-5p, hsa-miR-93-5p and hsa-miR-106b-3p) downregulated in sCJD and provided a combined AUC of 0.788^44^. In comparison, hsa-miR-16-5p was also found deregulated in our study however, upregulated in serum EVs of sCJD patients and pre-clinical M1000 infected mice. The difference in the direction of expression may be due to the enrichment of disease-associated miRNAs in EVs compared to those found in whole blood and circulating serum^16^. Similar to the study performed by Norsworthy et al.^44^, we did not find any correlation with miRNA expression and polymorphism subtypes. The enrichment of disease associated miRNAs causes an upregulation of miRNA compared to controls however, may be downregulated due to being diluted within the bloodstream when analysing non-EV samples. Nonetheless, the above recent studies^43,44^ show that there is interest in developing a blood-based test for sCJD, in particular, to screen suspected CJD cases. Combining the miRNA signatures found between our studies may help in refining a sCJD panel that can be applied to several cohorts and improve independent validation studies.

Currently, detection of 14-3-3 protein in cerebrospinal fluid (CSF) is widely performed together with EEGs to diagnosis suspected sCJD cases. The 14-3-3 proteins are a group of highly conserved proteins found in the brain involved in the regulation of protein phosphorylation and mitogen-activated protein kinase pathways. The combination of 14-3-3 and EEG have shown the ability to diagnose sCJD with 85% accuracy in a large study of data collected from 1572 autopsies by the CJD National Reference Centre register^45^. Magnetic resonance imaging (MRI) has also been an ideal diagnostic imaging method to observe prion-related lesions in the brain. Abnormalities in sCJD patients were confirmed in 83% of patients with a specificity of 83% and have formed part of routine testing protocols for sCJD^21^. New diagnostic criterion for MRI was recently updated upon examining 619 patients with sCJD and other controls which saw improved sensitivity between 90 and 95% as examined by four neuroradiologists^46^. Protein misfolding cyclic amplification assay (PMCA) and real-time quaking-induced prion conversion (RT-QuIC) which amplifies the presence of pathological prion PrP^Sc^ in a sample, such as CSF, can provide 82–96% sensitivity and 100% specificity^47,48^. Unfortunately, in this study, not all participants underwent both CSF tau and/or 14-3-3 testing in order to provide enough power to perform any correlation analysis with miRNA expression.

Neurofilament light chain (NFL), the main components of the neuronal cytoskeleton, have shown some diagnostic potential in human prion diseases and other neurogenerative diseases. The diagnostic value of NFL in CSF has seen 98% sensitivity and 65% specificity in the discrimination of sCJD from non-CJD cases^49^ and 100% sensitivity and 85.5% specificity in another study^50^. The field has extensively studied and validated the use of CSF samples for CJD diagnosis however, the use of blood samples, a relatively less-invasive specimen, is still in its infancy. Furthermore, the advantage of using qRT-PCR as a diagnostic tool allows for integration into current clinical workflows and relatively faster diagnostic reporting compared to the above methods. In addition, there is minimal biocontainment issues while using qRT-PCR compared to RT-QuIC which involves amplifying potentially infectious proteins.

The establishment of this study has provided additional insight into the miRNA expression changes associated with pre-clinical prion disease and potential serum EV biomarkers that can be used as a liquid biopsy of the brain. The longitudinal analysis performed in this mice study allows the potential to provide staging of disease progression in patients with a longer clinical duration such as CJD cases with a VV or MV phenotype. In addition, the biomarkers identified here can be used as a differential panel of miRNAs associated with other progressive dementias to diagnose suspected CJD cases and other neurodegenerative conditions^8^. The key findings from this study will thus facilitate the discovery of therapeutic and screening strategies of prion infectivity in biological specimens and enable the detection of pre-symptomatic sporadic prion diseases.

Prion disease is an infectious neurodegenerative disease that causes brain damage and severe memory impairment. EVs, secreted by cells, assist in the spread of infectious prions through the transfer of nucleic acid contents from cell to cell within various organs such as the brain. These biological vesicles also circulate in the bloodstream and can be captured to identify diagnostic indicators of disease. The type of biological indicator analysed in this study are short RNA transcripts called miRNA which regulate the expression of genes. miRNA changes can be observed before the appearance of pathological changes thus allowing the potential to find pre-clinical disease indicators. We used a mouse-adapted model of human prion disease to understand miRNA changes in the brain across the course of prion infection. We also analysed the circulating miRNA contained in extracellular vesicles within the blood to determine whether there is clinical utility of miRNA biomarkers during pre-clinical and clinical disease. The study revealed a collection of miRNAs that are different during the development of prion disease compared to uninfected animals. We then used the prion-associated miRNA biomarkers identified in the prion mouse model to screen human clinical samples which will benefit the development of a diagnostic test to detect prion-related diseases such as Creutzfeldt-Jakob disease.

## Methods

### Prion M1000 infected mouse model

All animals used in this study were approved by the University of Melbourne Animal Experimentation Ethics Committee (ID: 1111949). The animal experimental design was performed as previously published^11^. Six to eight-week-old balb/c mice (ARC) were anaesthetized with methoxyflurane and inoculated in the left parietal region with a 1% w/v brain homogenate prepared in PBS (Gibco) from the brain of a balb/c mouse with terminal M1000-induced prion disease (M1000 infected)^12^ or normal brain prepared from an uninfected balb/c mouse (uninfected)^3^. All mice were female as balb/c female mice have been shown to be a robust model for the study of mouse-adapted human prion strains. Animals were sacrificed under methoxyflurane anaesthesia at predetermined time points (3 and 13 wpi) or when signs consistent with terminal prion disease were evident, such as bradykinesia, weight loss, kyphosis, hind limb paresis and ataxia^3^. Wpi and standard deviation of the terminal uninfected mice was 20.21 weeks (SD: 1.47) and M1000 mice was 20.31 weeks (SD: 1.53)(Supplementary Table 1). Cardiac puncture bleed was performed on mice (*n* = 4–5) at each time point into MiniCollect 0.5 ml Z Serum Gel Separation tubes (Greiner Bio One), centrifuged at 3000 × *g* for 10 min at 4 °C before transferring serum into a new tube for RNA extraction using the Plasma/serum exosomal RNA isolation kit (Norgen Biotek, Canada) according to the manufacturer’s instructions. Any serum sample showing a greater haemolysis index of 20^51^ was rejected. The plasma/serum circulating and exosomal RNA isolation kit was previously demonstrated to produce similar miRNA profiles to those obtained from characterised exosomes isolated from serum^16^. In this study, these ‘exosomal’ RNA serum samples will be defined as ‘EV RNA/miRNA’ as no additional methods were used to further characterise the EVs collected as the kit used isolates ‘exosomal’ RNA and not whole EV/exosomes. Brains were removed at each time point and dissected into half along the midline of the brain. The thalamus region was dissected for analysis as previously described elsewhere^52,53^. Some brains were allocated for histology staining with hematoxylin and eosin using standard procedures and immunohistochemistry was performed with ICSM-18^11^. Each hemi-brain was flash-frozen in liquid nitrogen for western immunoblotting (using standard procedures with the SAF-32 anti-PrP antibody) while the other hemi-brain was placed in RNAlater (Life Technologies, Melbourne, VIC, Australia) to preserve RNA for isolation via the miRNeasy mini Kit (Qiagen, Chadstone, Australia) according to the manufacturer’s instructions.

### Small RNA sequencing and bioinformatics analysis

Extracted RNA from mice tissues and serum EVs were evaluated by using an Agilent 2100 Bioanalyser (Agilent Technologies, Palo Alto, CA, USA) and the RNA6000 and small RNA assays. Mice tissues were checked for RNA integrity and those with a RIN greater than 7 proceeded to library construction. For each small RNA library, RNA was ligated to adapters and barcodes to allow libraries to be pooled during sequencing. All libraries were constructed according to the manufacturer’s protocol (Ion Total RNA-seq kit v2, Life Technologies) and sequenced on the Ion Torrent PGM™ (Life Technologies) using Ion™ 318 Chips (Life Technologies) and the Ion PGM™ 200 Sequencing Kit (Life Technologies) as previously published^16^. Raw sequences from samples collected from the mouse study were subjected to pre-processing and sequence alignment using the default parameters provided by Torrent Suite™ v4.0 software (Life Technologies). Briefly, low quality reads were filtered by 3′ quality trimming and adapter clipping. Within the Torrent Suite™, high quality reads were parsed for whole-genome sequence mapping to *Mus musculus* v10 (mm10) build of the mouse genome from University of California Santa Cruz database using Torrent Mapping Alignment Program (TMAP) with their default parameters and analysed using Partek Genomic Suite. A number of 1401 miRNAs were identified in the dataset across all timepoints. Reads were normalised to RPM and only abundant miRNA species (minimum > 10 RPM across all thalamus and serum EV samples) were used for downstream analysis such as differential expression and gene ontology (GO) analysis. The Database for Annotation, Visualisation and Integrated Discovery (DAVID) v6.7^54^ were used to investigate GO biological processes, molecular functions and localisations of the corresponding gene of interest. The miRNA pathway analysis was performed using the default parameters in DIANA miRPath and miRTarBase according to the developer’s recommendations. Circos is a Perl-based software package for visualising large-scale multi-sample genomic information in circular ideogram^55^. Circos v0.64 was selected to display positional relationships between data track intervals.

### Validation of human clinical samples using qRT-PCR

All serum samples were collected by the Clinical Dementia Centre Göttingen. All patients provided written informed consent or their legal next of kin and approved from the local Ethics committee of the University Hospital of Göttingen as previously published^19^. Patients with sCJD (*n* = 26) were diagnosed according to established criteria^20,21^ and compared to controls (*n* = 20) in the discovery cohort. In the validation cohort, another 26 patients were recruited (sCJD, *n* = 20 and controls, *n* = 6). Each subject had undergone molecular genotyping to determine codon 129 subtyping (MM, MV or VV). Some subjects (11–15 subjects) also had additional testing performed including CSF Tau, CSF pTau, 14-3-3 Western blotting or ELISA or/and PrP^SC^ RT-QuIC to reach a diagnosis. Serum EV RNA was isolated by using the Plasma/serum circulating and exosomal RNA isolation kit (Norgen Biotek) from 500 μl serum per participant whereby the manufacturer’s protocol was followed. The yield of EV RNA was analysed by a small RNA assay and ran on the Agilent 2100 Bioanalyser (Agilent Technologies, Palo Alto, CA, USA) before cDNA conversion and qRT-PCR as previously published^8^. All data qRT-PCR assays were run with hsa-miR-185-5p, hsa-miR-451a and hsa-miR-93-5p as endogenous controls (identified to be consistently expressed across human serum EV samples by NGS and analysed by GENorm in our previous publications^8,16^), using the ViiA 7 software v1.2.2 (Life Technologies). All qRT-PCR was performed using the following Taqman primers (Life Technologies): hsa-miR-185-5p(Cat# 4427975; Assay ID:002271), hsa-miR-451a (Cat# 4427975; Assay ID:001141), hsa-miR-93-5p (Cat# 4427975; Assay ID:001090), hsa-miR-10b-5p (Cat# 4427975; Assay ID:002218), hsa-miR-133a-3p (Cat# 4427975; Assay ID:002246), hsa-miR-142-5p (Cat# 4427975; Assay ID:002248), hsa-miR-181a-5p (Cat# 4427975; Assay ID:000480), hsa-miR-423-3p (Cat# 4427975; Assay ID:002626), hsa-miR-101-3p (Cat# 4427975; Assay ID:002253), hsa-miR-146a-5p (Cat# 4427975; Assay ID:000468), hsa-miR-16-5p (Cat# 4427975, Assay ID:000391), hsa-miR-222-3p (Cat# 4427975; Assay ID:002276), hsa-miR-1306-5p (Cat# 4427975; Assay ID:242734_mat), hsa-miR-130a-3p (Cat# 4427975; Assay ID:000454), hsa-miR-148b-3p (Cat# 4427975; Assay ID:000471), hsa-miR-92a-3p (Cat# 4427975, Assay ID:000431), hsa-miR-142-3p (Cat# 4427975; Assay ID:000464).

### Statistics and reproducibility

Biostatistical analysis was performed in RStudio (Version 3.5.0) using the packages glmnet, randomForest and mplot using the Delta Ct values obtained from the qRT-PCR validation of human clinical samples. The cross-validation performance of the LASSO (least absolute shrinkage and selection operator) models and random forest models were accessed based on 20-fold experiments to make full use of our data. Finally, LASSO model was found to be superior to the random forest model. LASSO model was then trained on the 40 participants collected to select optimal weighting coefficients via penalised maximum likelihood to build a diagnostic miRNA signature. The regression analysis performed both variable selection and regularization in order to enhance the prediction accuracy and interpretability of the statistical model. Area under the curve (AUC) was employed to demonstrate the sensitivity and specificity of the four strongest miRNAs in predicting diagnosis. The mplot package was then used to provide an easy to use implementation of model stability and variable inclusion plots. For statistical analysis and reproducibility, in the mice study, 4–5 mice were used per group at each timepoint. For the human clinical validation study, *n* = 26 sCJD patients and *n* = 20 controls were used for the training cohort followed by another *n* = 20 sCJD and *n* = 6 controls for the validation test. Statistical hypothesis tests were conducted using all samples in each study.

### Reporting summary

Further information on research design is available in the Nature Research Reporting Summary linked to this article.

## Supplementary information

Supplementary Information

Description of Additional Supplementary Files

Supplementary Data 1

Reporting Summary

## Data Availability

Small RNA sequencing data that support the findings of this study have been deposited in the European Nucleotide Archive with accession number PRJEB42021. All relevant data are available from the authors upon request to the corresponding author. Source data underlying plots shown in figures are provided in Supplementary Data 1.
