# Supplementary material for: Distribution of microRNA profiles in pre-clinical and clinical forms of murine and human prion disease
Source: Commun Biol. 2021 Mar 25;4:411. doi: 10.1038/s42003-021-01868-x (PMC7994852; doi:10.1038/s42003-021-01868-x)

Supplementary Tables

Supplementary Table 1 – Details of mice used in the study

Supplementary Table 2 - Most abundant miRNAs found in the thalamus

Supplementary Table 3 - Differentially expressed miRNA identified in the thalamus

Supplementary Table 4 - Most abundant miRNAs expressed in the serum

Supplementary Table 5 - Differentially expressed miRNA identified in the serum

Supplementary Table 6 - Differentially expressed miRNA candidates selected for validation study

Supplementary Figures

Supplementary Figure 1 – Uncropped western blot of Figure 1B.

Supplementary Figure 2 – PCA plots of small RNA sequencing performed on thalamus and serum EV samples

Supplementary Table 1 – Details of mice used in the study

Mice #	Timepoint	Condition	N number	Cull date (Wpi)	RIN*	Thalamus	Serum EVs
1	Week 3	Uninfected	1	3	8.1	Y	Y
2	Week 3	Uninfected	2	3	8.3	Y	Y
3	Week 3	Uninfected	3	3	7.9	Y	Y
4	Week 3	Uninfected	4	3	8.9	Y	Y
5	Week 3	Uninfected	5	3	8.4	Y	Y
6	Week 3	Uninfected	6	3	N/A	N	Y
7	Week 3	M1000	1	3	8.5	Y	Y
8	Week 3	M1000	2	3	7.6	Y	Y
9	Week 3	M1000	3	3	8.6	Y	Y
10	Week 3	M1000	4	3	8.9	Y	Y
11	Week 3	M1000	5	3	N/A	N	Y
12	Week 3	M1000	6	3	N/A	N	Y
13	Week 13	Uninfected	1	13	9	Y	Y
14	Week 13	Uninfected	2	13	8.8	Y	Y
15	Week 13	Uninfected	3	13	9.1	Y	Y
16	Week 13	Uninfected	4	13	8.5	Y	Y
17	Week 13	Uninfected	5	13	8.9	Y	Y
18	Week 13	Uninfected	6	13	N/A	N	Y
19	Week 13	M1000	1	13	7.9	Y	Y
20	Week 13	M1000	2	13	7.7	Y	Y
21	Week 13	M1000	3	13	7.9	Y	Y
22	Week 13	M1000	4	13	7.4	Y	Y
23	Week 13	M1000	5	13	N/A	N	Y
24	Terminal	Uninfected	1	18.4	8.5	Y	Y
25	Terminal	Uninfected	2	18.4	9.8	Y	Y
26	Terminal	Uninfected	3	21.4	8.1	Y	Y
27	Terminal	Uninfected	4	21.4	8.6	Y	Y
28	Terminal	Uninfected	5	20.1	8.7	Y	Y
29	Terminal	Uninfected	6	21.4	N/A	N	Y
30	Terminal	M1000	1	18	7.1	Y	N
31	Terminal	M1000	2	20	7.9	Y	Y
32	Terminal	M1000	3	21.5	7.7	Y	Y
33	Terminal	M1000	4	22	8.1	Y	Y
34	Terminal	M1000	5	18	8.3	Y	Y
35	Terminal	M1000	6	21	N/A	N	Y

Wpi = week post infection, RIN = RNA integrity number
All mice were female

Supplementary Table 2 – Most abundant miRNAs found in the thalamus

Most abundant miRNA species in the thalmaus			
mmu-let-7a-5p	mmu-miR-16-5p	mmu-miR-31-5p	mmu-miR-451a
mmu-let-7b-3p	mmu-miR-17-5p	mmu-miR-3102-3p	mmu-miR-455-3p
mmu-let-7b-5p	mmu-miR-181a-1-3p	mmu-miR-320-3p	mmu-miR-484
mmu-let-7c-5p	mmu-miR-181a-5p	mmu-miR-323-3p	mmu-miR-486a-5p
mmu-let-7d-3p	mmu-miR-181b-5p	mmu-miR-324-5p	mmu-miR-486b-5p
mmu-let-7d-5p	mmu-miR-181c-3p	mmu-miR-326-3p	mmu-miR-487b-3p
mmu-let-7e-5p	mmu-miR-181c-5p	mmu-miR-328-3p	mmu-miR-495-3p
mmu-let-7f-5p	mmu-miR-1839-3p	mmu-miR-329-3p	mmu-miR-497a-5p
mmu-let-7g-5p	mmu-miR-1839-5p	mmu-miR-329-5p	mmu-miR-500-3p
mmu-let-7i-5p	mmu-miR-185-5p	mmu-miR-330-3p	mmu-miR-505-5p
mmu-miR-100-5p	mmu-miR-187-3p	mmu-miR-330-5p	mmu-miR-532-5p
mmu-miR-101a-3p	mmu-miR-18a-5p	mmu-miR-331-3p	mmu-miR-541-5p
mmu-miR-103-3p	mmu-miR-191-5p	mmu-miR-335-5p	mmu-miR-543-3p
mmu-miR-106b-5p	mmu-miR-192-5p	mmu-miR-337-5p	mmu-miR-551b-3p
mmu-miR-107-3p	mmu-miR-193b-3p	mmu-miR-338-3p	mmu-miR-574-3p
mmu-miR-1224-3p	mmu-miR-195a-5p	mmu-miR-338-5p	mmu-miR-652-3p
mmu-miR-1224-5p	mmu-miR-1981-5p	mmu-miR-340-3p	mmu-miR-664-3p
mmu-miR-124-3p	mmu-miR-19a-3p	mmu-miR-340-5p	mmu-miR-667-3p
mmu-miR-124-5p	mmu-miR-19b-3p	mmu-miR-342-3p	mmu-miR-668-3p
mmu-miR-125a-5p	mmu-miR-1a-3p	mmu-miR-344-3p	mmu-miR-669a-3p
mmu-miR-125b-2-3p	mmu-miR-203-3p	mmu-miR-344d-3p	mmu-miR-671-5p
mmu-miR-125b-5p	mmu-miR-204-3p	mmu-miR-345-5p	mmu-miR-672-5p
mmu-miR-126a-3p	mmu-miR-204-5p	mmu-miR-346-5p	mmu-miR-674-3p
mmu-miR-126a-5p	mmu-miR-20a-5p	mmu-miR-34a-5p	mmu-miR-674-5p
mmu-miR-127-3p	mmu-miR-212-3p	mmu-miR-34b-3p	mmu-miR-700-3p
mmu-miR-127-5p	mmu-miR-218-5p	mmu-miR-34b-5p	mmu-miR-700-5p
mmu-miR-128-3p	mmu-miR-219a-2-3p	mmu-miR-34c-5p	mmu-miR-708-5p
mmu-miR-129-1-3p	mmu-miR-21a-5p	mmu-miR-3547-3p	mmu-miR-744-5p
mmu-miR-129-2-3p	mmu-miR-22-3p	mmu-miR-361-3p	mmu-miR-770-3p
mmu-miR-129-5p	mmu-miR-22-5p	mmu-miR-361-5p	mmu-miR-7a-1-3p
mmu-miR-130a-3p	mmu-miR-221-3p	mmu-miR-365-3p	mmu-miR-7a-2-3p
mmu-miR-132-3p	mmu-miR-222-3p	mmu-miR-369-3p	mmu-miR-7a-5p
mmu-miR-132-5p	mmu-miR-223-3p	mmu-miR-369-5p	mmu-miR-7b-5p
mmu-miR-133a-3p	mmu-miR-23a-3p	mmu-miR-370-3p	mmu-miR-872-3p
mmu-miR-134-5p	mmu-miR-23b-3p	mmu-miR-374b-5p	mmu-miR-872-5p
mmu-miR-135a-5p	mmu-miR-24-3p	mmu-miR-376a-3p	mmu-miR-873a-3p
mmu-miR-135b-5p	mmu-miR-26a-5p	mmu-miR-376a-5p	mmu-miR-873a-5p
mmu-miR-136-3p	mmu-miR-26b-5p	mmu-miR-376b-3p	mmu-miR-9-3p
mmu-miR-136-5p	mmu-miR-27a-3p	mmu-miR-376b-5p	mmu-miR-9-5p
mmu-miR-137-3p	mmu-miR-27b-3p	mmu-miR-376c-3p	mmu-miR-92b-3p
mmu-miR-138-2-3p	mmu-miR-298-5p	mmu-miR-378a-3p	mmu-miR-93-5p
mmu-miR-138-5p	mmu-miR-29a-3p	mmu-miR-379-5p	mmu-miR-935
mmu-miR-139-5p	mmu-miR-29b-2-5p	mmu-miR-381-3p	mmu-miR-98-5p
mmu-miR-140-3p	mmu-miR-29b-3p	mmu-miR-382-5p	mmu-miR-99a-5p
mmu-miR-140-5p	mmu-miR-29c-3p	mmu-miR-383-5p	mmu-miR-99b-5p
mmu-miR-142a-3p	mmu-miR-29c-5p	mmu-miR-384-3p	

mmu-miR-143-3p	mmu-miR-300-3p	mmu-miR-384-5p
mmu-miR-144-3p	mmu-miR-301a-3p	mmu-miR-409-3p
mmu-miR-144-5p	mmu-miR-3068-3p	mmu-miR-409-5p
mmu-miR-145a-5p	mmu-miR-3068-5p	mmu-miR-410-3p
mmu-miR-148a-3p	mmu-miR-3071-3p	mmu-miR-411-3p
mmu-miR-149-5p	mmu-miR-30a-3p	mmu-miR-411-5p
mmu-miR-150-5p	mmu-miR-30a-5p	mmu-miR-421-3p
mmu-miR-151-5p	mmu-miR-30b-5p	mmu-miR-425-5p
mmu-miR-152-3p	mmu-miR-30c-5p	mmu-miR-433-3p
mmu-miR-153-3p	mmu-miR-30d-5p	mmu-miR-434-3p
mmu-miR-154-5p	mmu-miR-30e-3p	mmu-miR-434-5p
mmu-miR-15a-5p	mmu-miR-30e-5p	mmu-miR-448-5p

** Minimum reads all samples >10 RPM*

Supplementary Table 3 - Differentially expressed miRNA identified in the thalamus

Biomarker	***Time point***	***Week 3 preclinical stage***		***Week 13 pre-clinical stage***		***Terminal stage***	
		Thalamus		Thalamus		Thalamus	
		p-value	FC*	p-value	FC*	p-value	FC*
mmu-miR-129-1-3p	W3	**0.00186592**	**-6.05479**	0.836759	1.21333	0.283836	-2.01164
mmu-miR-129b-5p	W3	**0.00100516**	**-2.62686**	0.664837	1.17964	0.732312	-1.13505
mmu-miR-337-3p	W3	**0.0459807**	**1.93411**	0.545386	1.23682	0.618055	-1.1515
mmu-let-7c-2-3p	W13	0.834378	1.08133	**0.0394445**	**2.27756**	0.756139	-1.07419
mmu-miR-133a-3p	W13	0.819566	1.12466	**0.0351433**	**1.9848**	0.165813	-1.46817
mmu-miR-181a-5p	W13	0.860873	-1.04241	**0.0401921**	**1.62435**	0.568114	-1.09513
mmu-miR-1a-3p	W13	0.987852	-1.00663	**0.0360615**	**1.99904**	0.075089	-1.48795
mmu-miR-30a-3p	W13	0.871893	-1.05271	**0.0230981**	**1.98994**	0.41294	-1.17071
mmu-miR-3102-3p	W13	0.713974	-1.07177	**0.0428382**	**1.52264**	0.268337	1.17238
mmu-miR-365-3p	W13	0.185391	2.36191	**0.049061**	**2.4518**	0.995738	-1.00142
mmu-miR-455-3p	W13	0.806189	-1.08239	**0.0114005**	**2.48201**	0.160263	1.29164
mmu-miR-505-5p	W13	0.0714303	1.39545	**0.036753**	**1.65756**	0.693389	1.06773
mmu-miR-6240	W13	0.597216	1.2031	**0.046904**	**1.98721**	0.470447	1.29224
mmu-miR-328-3p	W13/T	0.77288	1.06029	**0.00409319**	**1.8754**	0.022016	1.34896
mmu-miR-101-3p	W13/T	0.381466	1.2964	**0.494949**	**1.63994**	**0.0143**	**-1.53798**
mmu-miR-10a-5p	T	0.260386	-2.10311	0.103402	1.87299	**0.026127**	**-1.75153**
mmu-miR-142-3p	T	0.931816	-1.04862	0.661625	1.2986	**0.001175**	**2.48518**
mmu-miR-223-3p	T	0.772433	-1.14881	0.915772	-1.04597	**0.028097**	**1.71737**
mmu-miR-296-5p	T	0.62607	-1.37237	0.117833	2.16411	**0.015471**	**2.46612**
mmu-miR-370-3p	T	0.681218	1.14312	0.613993	1.22356	**0.013459**	**1.85792**

*Prion infected (M1000) Vs control (uninfected)

Bold values are significant at time point

Supplementary Table 4 – Most abundant miRNAs expressed in the serum

**Most abundant miRNA species in serum EVs**			
mmu-let-7a-5p	mmu-miR-144-3p	mmu-miR-210-3p	mmu-miR-340-5p
mmu-let-7b-5p	mmu-miR-144-5p	mmu-miR-214-3p	mmu-miR-342-3p
mmu-let-7c-5p	mmu-miR-145a-5p	mmu-miR-21a-5p	mmu-miR-34a-5p
mmu-let-7d-3p	mmu-miR-146a-5p	mmu-miR-22-3p	mmu-miR-361-5p
mmu-let-7d-5p	mmu-miR-148a-3p	mmu-miR-22-5p	mmu-miR-374b-5p
mmu-let-7f-5p	mmu-miR-150-5p	mmu-miR-221-3p	mmu-miR-378a-3p
mmu-let-7g-5p	mmu-miR-151-5p	mmu-miR-222-3p	mmu-miR-378a-5p
mmu-let-7i-5p	mmu-miR-152-3p	mmu-miR-223-3p	mmu-miR-378c
mmu-miR-101a-3p	mmu-miR-15a-5p	mmu-miR-23a-3p	mmu-miR-421-3p
mmu-miR-103-3p	mmu-miR-15b-3p	mmu-miR-23b-3p	mmu-miR-423-3p
mmu-miR-106b-3p	mmu-miR-15b-5p	mmu-miR-24-3p	mmu-miR-423-5p
mmu-miR-106b-5p	mmu-miR-16-2-3p	mmu-miR-25-3p	mmu-miR-425-5p
mmu-miR-107-3p	mmu-miR-16-5p	mmu-miR-26a-5p	mmu-miR-451a
mmu-miR-10a-5p	mmu-miR-17-3p	mmu-miR-26b-5p	mmu-miR-484
mmu-miR-10b-5p	mmu-miR-17-5p	mmu-miR-27a-3p	mmu-miR-486a-3p
mmu-miR-1198-5p	mmu-miR-181a-5p	mmu-miR-27b-3p	mmu-miR-486a-5p
mmu-miR-122-5p	mmu-miR-185-5p	mmu-miR-29a-3p	mmu-miR-486b-5p
mmu-miR-124-3p	mmu-miR-186-5p	mmu-miR-29b-3p	mmu-miR-497a-5p
mmu-miR-125a-5p	mmu-miR-18a-5p	mmu-miR-29c-3p	mmu-miR-503-5p
mmu-miR-125b-5p	mmu-miR-191-5p	mmu-miR-301a-3p	mmu-miR-574-3p
mmu-miR-126a-3p	mmu-miR-192-5p	mmu-miR-30a-3p	mmu-miR-652-3p
mmu-miR-126a-5p	mmu-miR-194-5p	mmu-miR-30a-5p	mmu-miR-669a-3p
mmu-miR-128-3p	mmu-miR-195a-5p	mmu-miR-30b-5p	mmu-miR-674-5p
mmu-miR-130a-3p	mmu-miR-199a-3p	mmu-miR-30c-5p	mmu-miR-744-5p
mmu-miR-130b-3p	mmu-miR-199b-3p	mmu-miR-30d-5p	mmu-miR-7a-5p
mmu-miR-133a-3p	mmu-miR-19a-3p	mmu-miR-30e-5p	mmu-miR-92a-3p
mmu-miR-139-5p	mmu-miR-19b-3p	mmu-miR-31-5p	mmu-miR-93-5p
mmu-miR-140-3p	mmu-miR-1a-3p	mmu-miR-320-3p	mmu-miR-99a-5p
mmu-miR-140-5p	mmu-miR-200c-3p	mmu-miR-322-5p	mmu-miR-99b-5p
mmu-miR-142a-3p	mmu-miR-203-3p	mmu-miR-328-3p	
mmu-miR-142a-5p	mmu-miR-205-5p	mmu-miR-335-5p	
mmu-miR-143-3p	mmu-miR-20a-5p	mmu-miR-339-5p	

*Minimum reads all samples >10RPM

Supplementary Table 5 - Differentially expressed miRNA identified in the serum

Biomarker	Time point	Week 3 preclinical stage		Week 13 pre-clinical stage		Terminal stage	
		Serum		Serum		Serum	
		p-value	FC*	p-value	FC*	p-value	FC*
mmu-miR-1306-5p	W3	**0.0396902**	**1.56242**	0.323129	1.37987	0.951458	-1.01181
mmu-miR-130a-3p	W3	**0.0299279**	**1.58088**	0.266282	1.27904	0.511283	-1.12737
mmu-miR-148b-3p	W3	**0.0374105**	**1.90161**	0.575217	1.28044	0.121289	-1.65838
mmu-miR-92a-3p	W3	**0.0174073**	**1.51023**	0.431542	1.19898	0.834288	-1.02923
mmu-miR-142-3p	W3/T	**0.00842767**	**-1.53245**	0.459868	-1.22478	**0.000382**	**-1.8023**
mmu-miR-15b-5p	W3/T	**0.0499997**	**1.47179**	0.327648	1.4156	**0.022705**	**-1.69291**
mmu-miR-181a-5p	W13	0.7647	1.20182	**0.018231**	**-3.11036**	0.358598	-1.48811
mmu-miR-1a-3p	W13	0.793019	-1.08966	**0.016156**	**2.44338**	0.707372	1.14393
mmu-miR-223-5p	W13	0.212399	-2.40739	**0.010029**	**2.31205**	0.422192	-1.29238
mmu-miR-423-3p	W13	0.603742	1.16111	**0.026073**	**-1.79527**	0.911576	1.01964
mmu-miR-146a-5p	W13/T	0.459056	-1.2149	**0.00058**	**-1.87395**	**0.000425**	**-1.82863**
mmu-miR-16-5p	W13/T	0.0857583	1.34601	**0.048308**	**1.84017**	**0.000307**	**-2.11812**
mmu-miR-205-5p	W13/T	0.624185	1.14256	**0.007889**	**-1.66194**	**0.0001**	**-2.21353**
mmu-miR-222-3p	W13/T	0.943246	1.0296	**0.009048**	**-2.90388**	**0.00727**	**-2.40001**
mmu-miR-10b-5p	T	0.747919	-1.0871	0.322239	1.23515	**0.000307**	**-1.92841**
mmu-miR-133a-3p	T	0.899358	1.04169	0.672893	-1.09876	**0.018162**	**2.0274**
mmu-miR-142-5p	T	0.324333	1.19207	0.733015	1.12464	**0.029399**	**-1.45771**
mmu-miR-203-3p	T	0.857682	-1.06741	0.218836	-1.36436	**0.000529**	**-2.16688**
mmu-miR-215-5p	T	0.306383	-1.46203	0.099677	-1.56508	**3.45E-05**	**-1.82609**

*Prion infected (M1000) Vs control (uninfected)

Bold values are significant at time point

Supplementary Table 6 - Differentially expressed miRNA candidates selected for validation study

Biomarker	Time point	Week 3 preclinical stage				Week 13 pre-clinical stage				Terminal stage			
		Serum		Thalamus		Serum		Thalamus		Serum		Thalamus	
		p-value	FC*	p-value	FC*	p-value	FC*	p-value	FC*	p-value	FC*	p-value	FC*
hsa-miR-185-5p	Control	N/A	N/A	N/A	N/A	N/A	N/A	N/A	N/A	N/A	N/A	N/A	N/A
hsa-miR-451a	Control	N/A	N/A	N/A	N/A	N/A	N/A	N/A	N/A	N/A	N/A	N/A	N/A
hsa-miR-93-5p	Control	N/A	N/A	N/A	N/A	N/A	N/A	N/A	N/A	N/A	N/A	N/A	N/A
hsa-miR-10b-5p	T	N/A	N/A	N/A	N/A	N/A	N/A	N/A	N/A	**0.00**	**-1.93**	0.23	-1.78
hsa-miR-133a-3p	T	N/A	N/A	N/A	N/A	N/A	N/A	N/A	N/A	**0.02**	**2.03**	0.16	-1.47
hsa-miR-142-5p	T	N/A	N/A	N/A	N/A	N/A	N/A	N/A	N/A	**0.03**	**-1.46**	0.90	1.42
hsa-miR-203a-3p	T	N/A	N/A	N/A	N/A	N/A	N/A	N/A	N/A	**0.00**	**-2.17**	0.19	1.33
hsa-miR-1a-3p	W13	N/A	N/A	N/A	N/A	**0.00**	**2.44**	**0.04**	**2.00**	N/A	N/A	N/A	N/A
hsa-miR-181a-5p	W13	N/A	N/A	N/A	N/A	**0.02**	**-3.11**	**0.04**	**1.62**	N/A	N/A	N/A	N/A
hsa-miR-423-3p	W13	N/A	N/A	N/A	N/A	**0.03**	**-1.80**	0.07	1.68	N/A	N/A	N/A	N/A
hsa-miR-101-3p	W13/T	N/A	N/A	N/A	N/A	0.75	1.50	**0.05**	**1.64**	0.73	-1.34	**0.01**	**-1.54**
hsa-miR-146a-5p	W13/T	N/A	N/A	N/A	N/A	**0.00**	**-1.87**	0.84	2.57	**0.00**	**-1.83**	0.24	6.18
hsa-miR-16-5p	W13/T	N/A	N/A	N/A	N/A	**0.05**	**1.84**	0.87	1.19	**0.00**	**-2.12**	0.79	-1.15
hsa-miR-205-5p	W13/T	N/A	N/A	N/A	N/A	**0.01**	**-1.66**	0.97	-1.42	**0.00**	**-2.21**	0.99	-1.26
hsa-miR-222-3p	W13/T	N/A	N/A	N/A	N/A	**0.01**	**-2.90**	0.83	2.20	**0.01**	**-2.40**	0.97	-1.09
hsa-miR-1306-5p	W3	**0.04**	**1.56**	0.49	-1.52	N/A	N/A	N/A	N/A	N/A	N/A	N/A	N/A
hsa-miR-130a-3p	W3	**0.03**	**1.58**	0.37	1.39	N/A	N/A	N/A	N/A	N/A	N/A	N/A	N/A
hsa-miR-148b-3p	W3	**0.04**	**1.90**	0.41	-1.24	N/A	N/A	N/A	N/A	N/A	N/A	N/A	N/A
hsa-miR-92a-3p	W3	**0.02**	**1.51**	1.00	-1.00	N/A	N/A	N/A	N/A	N/A	N/A	N/A	N/A
hsa-miR-142-3p	W3/T	**0.01**	**-1.53**	0.95	-1.05	N/A	N/A	N/A	N/A	**0.00**	**-1.80**	**0.00**	**2.49**

*Prion infected (M1000) Vs control (uninfected)
Bold values are significant at time point

Supplementary Figure 1 – Uncropped western blot of Figure 1B.

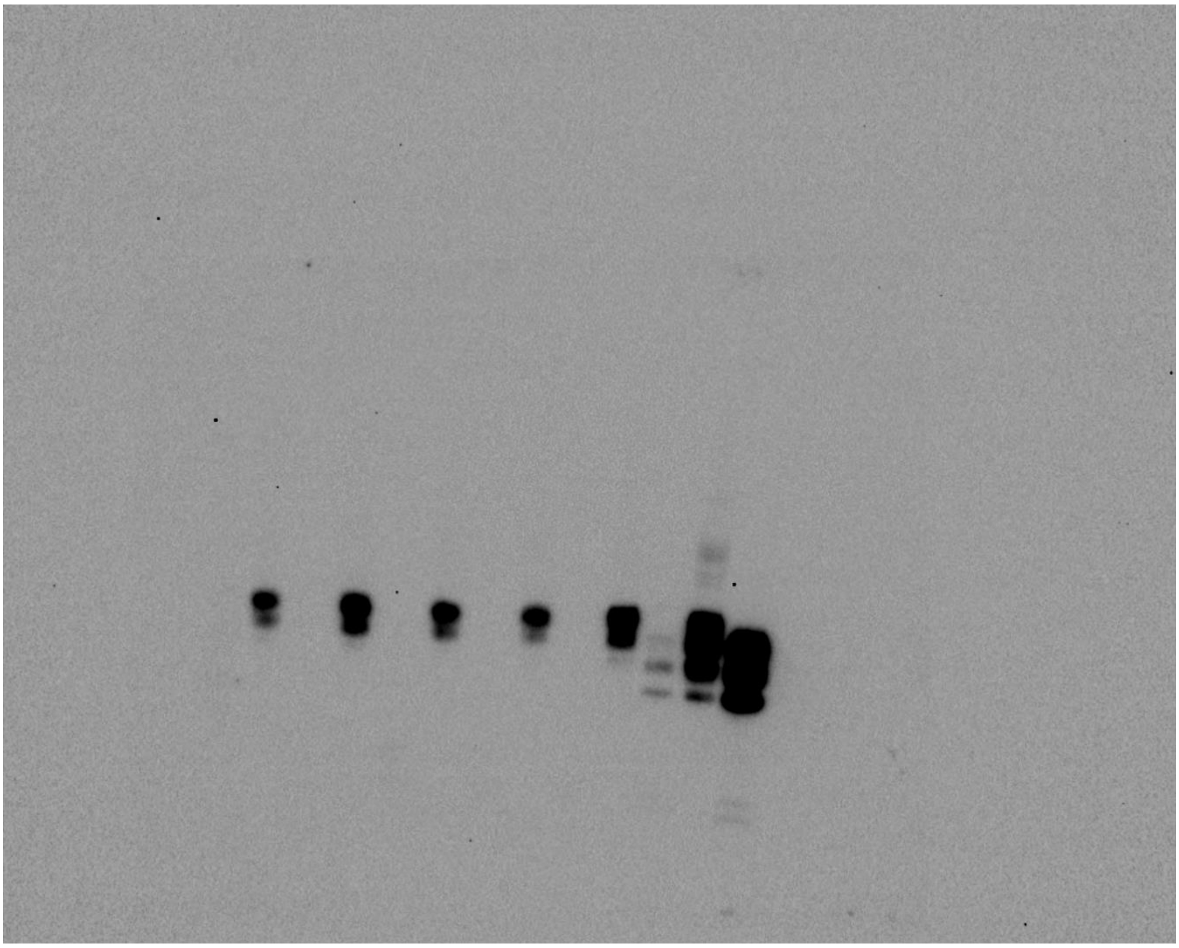

Supplementary Figure 2 - PCA plots of small RNA sequencing performed on thalamus and serum EV samples

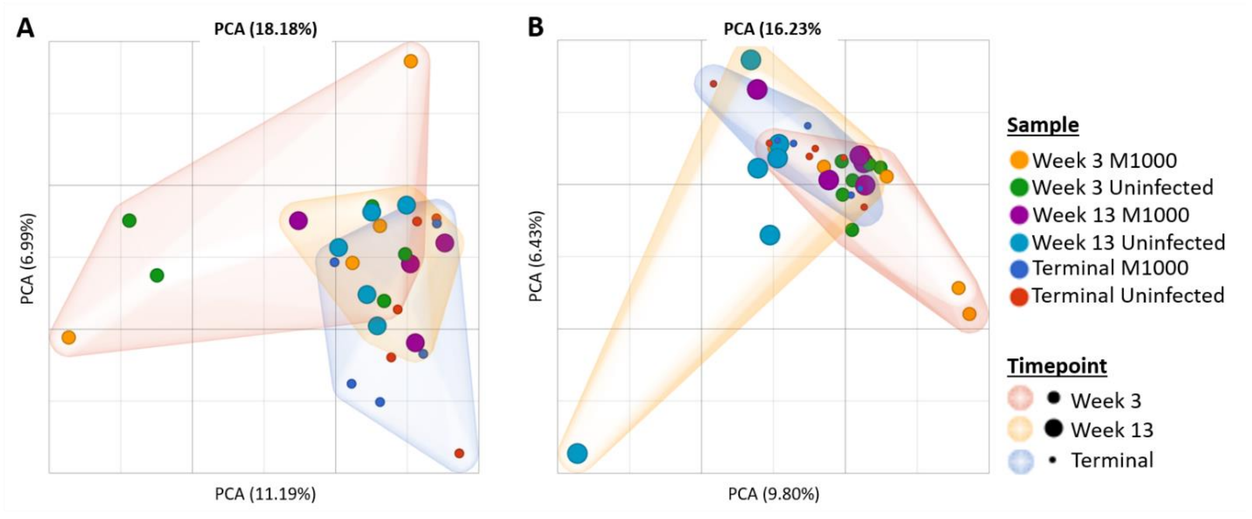

Supplement: Supplementary file 1 — Supplementary Information [file 42003_2021_1868_MOESM1_ESM.pdf]
