# Supplementary material for: Distribution of microRNA profiles in pre-clinical and clinical forms of murine and human prion disease
Source: Commun Biol. 2021 Mar 25;4:411. doi: 10.1038/s42003-021-01868-x (PMC7994852; doi:10.1038/s42003-021-01868-x)
Supplement: Supplementary file 4 — Reporting Summary [file 42003_2021_1868_MOESM4_ESM.pdf]

nature research

Corresponding author(s): Lesley Cheng
Andrew F. Hill

Last updated by author(s): Feb 19, 2021

Reporting Summary

Nature Research wishes to improve the reproducibility of the work that we publish. This form provides structure for consistency and transparency in reporting. For further information on Nature Research policies, see our Editorial Policies and the Editorial Policy Checklist.

Statistics

For all statistical analyses, confirm that the following items are present in the figure legend, table legend, main text, or Methods section.

n/a	Confirmed	
☐	☒	The exact sample size (*n*) for each experimental group/condition, given as a discrete number and unit of measurement
☐	☒	A statement on whether measurements were taken from distinct samples or whether the same sample was measured repeatedly
☐	☒	The statistical test(s) used AND whether they are one- or two-sided<br>*Only common tests should be described solely by name; describe more complex techniques in the Methods section.*
☐	☒	A description of all covariates tested
☐	☒	A description of any assumptions or corrections, such as tests of normality and adjustment for multiple comparisons
☐	☒	A full description of the statistical parameters including central tendency (e.g. means) or other basic estimates (e.g. regression coefficient) AND variation (e.g. standard deviation) or associated estimates of uncertainty (e.g. confidence intervals)
☒	☐	For null hypothesis testing, the test statistic (e.g. *F*, *t*, *r*) with confidence intervals, effect sizes, degrees of freedom and *P* value noted<br>*Give P values as exact values whenever suitable.*
☒	☐	For Bayesian analysis, information on the choice of priors and Markov chain Monte Carlo settings
☒	☐	For hierarchical and complex designs, identification of the appropriate level for tests and full reporting of outcomes
☒	☐	Estimates of effect sizes (e.g. Cohen's *d*, Pearson's *r*), indicating how they were calculated

Our web collection on statistics for biologists contains articles on many of the points above.

Software and code

Policy information about availability of computer code

Data collection	Torrent Suite v4.0
Data analysis	The Database for Annotation, Visualisation and Integrated Discovery (DAVID) v6.7 were used to investigate GO biological processes, molecular functions and localisations of the corresponding gene of interest. The mi RNA pathway analysis was performed using the default parameters in DIANA miRPath and miRTarBase according to developer's recommendations. Circos v0.64 was selected to display positional relationships between data track intervals.

For manuscripts utilizing custom algorithms or software that are central to the research but not yet described in published literature, software must be made available to editors and reviewers. We strongly encourage code deposition in a community repository (e.g. GitHub). See the Nature Research guidelines for submitting code & software for further information.

Data

Policy information about availability of data

All manuscripts must include a data availability statement. This statement should provide the following information, where applicable:

- Accession codes, unique identifiers, or web links for publicly available datasets
- A list of figures that have associated raw data
- A description of any restrictions on data availability

Small RNA sequencing data that support the findings of this study have been deposited in the European Nucleotide Archive with accession number PRJEB42021 (http://www.ebi.ac.uk/ena/data/view/PRJEB42021). All relevant data are available from the authors upon request to the corresponding author. Source data underlying plots shown in figures are provided in Supplementary Data 1.

Field-specific reporting

Please select the one below that is the best fit for your research. If you are not sure, read the appropriate sections before making your selection.

☒ Life sciences ☐ Behavioural & social sciences ☐ Ecological, evolutionary & environmental sciences

For a reference copy of the document with all sections, see nature.com/documents/nr-reporting-summary-flat.pdf

Life sciences study design

All studies must disclose on these points even when the disclosure is negative.

Sample size	For mouse experiments, for each timepoint n=4-5 was selected. Human samples were provided by a tissue bank and the available samples for analysis were provided (n=26 for CJD and n=20 for non-dementia).
Data exclusions	No data was excluded.
Replication	Next generation sequencing was performed to identify miRNA profiles associated with prion infection in mice (discovery). These were then analysed in human samples (validation)
Randomization	Randomisation was not relevant to this study. Discovery miRNA profiles were obtained from the animal model and used to identify profiles for testing in the human samples. A covariate of codon 129 PRNP genotype was used in analysis of the human samples.
Blinding	The investigators were not blinded to the analysis of the samples as this was a discovery data collection (animal model). For the human samples, a discovery set was used and further samples (CJD n=20; control n=6) for validation.

Reporting for specific materials, systems and methods

We require information from authors about some types of materials, experimental systems and methods used in many studies. Here, indicate whether each material, system or method listed is relevant to your study. If you are not sure if a list item applies to your research, read the appropriate section before selecting a response.

Materials & experimental systems

n/a	Involved in the study	
☐	☒	Antibodies
☒	☐	Eukaryotic cell lines
☒	☐	Palaeontology and archaeology
☐	☒	Animals and other organisms
☐	☒	Human research participants
☒	☐	Clinical data
☒	☐	Dual use research of concern

Methods

n/a	Involved in the study	
☒	☐	ChIP-seq
☒	☐	Flow cytometry
☒	☐	MRI-based neuroimaging

Antibodies

Antibodies used	R19.2 (polyclonal antibody against a peptide of amino acids 89-103 of the PrP sequence
Validation	Previously published (Vella et al, J Pathology 2007)

Animals and other organisms

Policy information about studies involving animals; ARRIVE guidelines recommended for reporting animal research

Laboratory animals	Balb/C wild-type mice. Female mice were used.
Wild animals	N/A
Field-collected samples	N/A
Ethics oversight	All animals used in this study were approved by the University of Melbourne Animal Experimentation Ethics Committee (ID: 1111949).

Note that full information on the approval of the study protocol must also be provided in the manuscript.

Human research participants

Policy information about studies involving human research participants

Population characteristics	All serum samples were collected by the Clinical Dementia Centre Goettingen. All patients provided written informed consent or their legal next of kin and approved from the local Ethics committee of the University Hospital of Goettingen.
Recruitment	Patients were from clinics related to neurodegenerative diseases such as prion disease. The samples had been diagnosed either as prion disease or non dementia.
Ethics oversight	Ethics committee of the University Hospital of Goettingen.

Note that full information on the approval of the study protocol must also be provided in the manuscript.
